# In memoriam Ching-I Peng (1950–2018)—an outstanding scientist and mentor with a remarkable legacy

**DOI:** 10.1186/s40529-020-00291-5

**Published:** 2020-04-25

**Authors:** Kuo-Fang Chung

**Affiliations:** Research Museum and Herbarium (HAST), Biodiversity Research Center, Academia Sinica, Taipei, Taiwan

## Abstract

Ching-I Peng, the most prolific and internationally recognized Taiwanese plant taxonomist of his generation, passed away on May 1, 2018. Dr. Peng was an eminent worker on the taxonomy of East Asian plants and the genus *Ludwigia*, and the foremost expert on Asian *Begonia*. He served as associate editor, co-editor in chief, and editor-in-chief of *Botanical Studies* and its predecessor *Botanical Bulletin of Academia Sinica* during the period 1992–2016. He gathered over 25,000 plant specimens, name 121 plant taxa, and has left a remarkable legacy of literature, collaborations and collections. This article summarizes Dr. Peng’s academic career and commemorates his enduring contribution.

In the late afternoon of May 1 2018, Dr. Ching-I Peng (彭鏡毅; Fig. 1) passed away at 68 years of age in the National Taiwan University Hospital after a year-long battle with acute myeloid leukemia. His unexpected early passing is a great loss for both the Taiwanese and the international botanical communities. Dr. Peng was the most productive and internationally recognized Taiwanese plant taxonomist of his generation (Table 1; Additional file 1). He was one of the foremost experts on the Taiwanese flora and the genus *Ludwigia*. During the last decade, he also became one of the most influential *Begonia* researchers in the world.

**Fig. 1:**
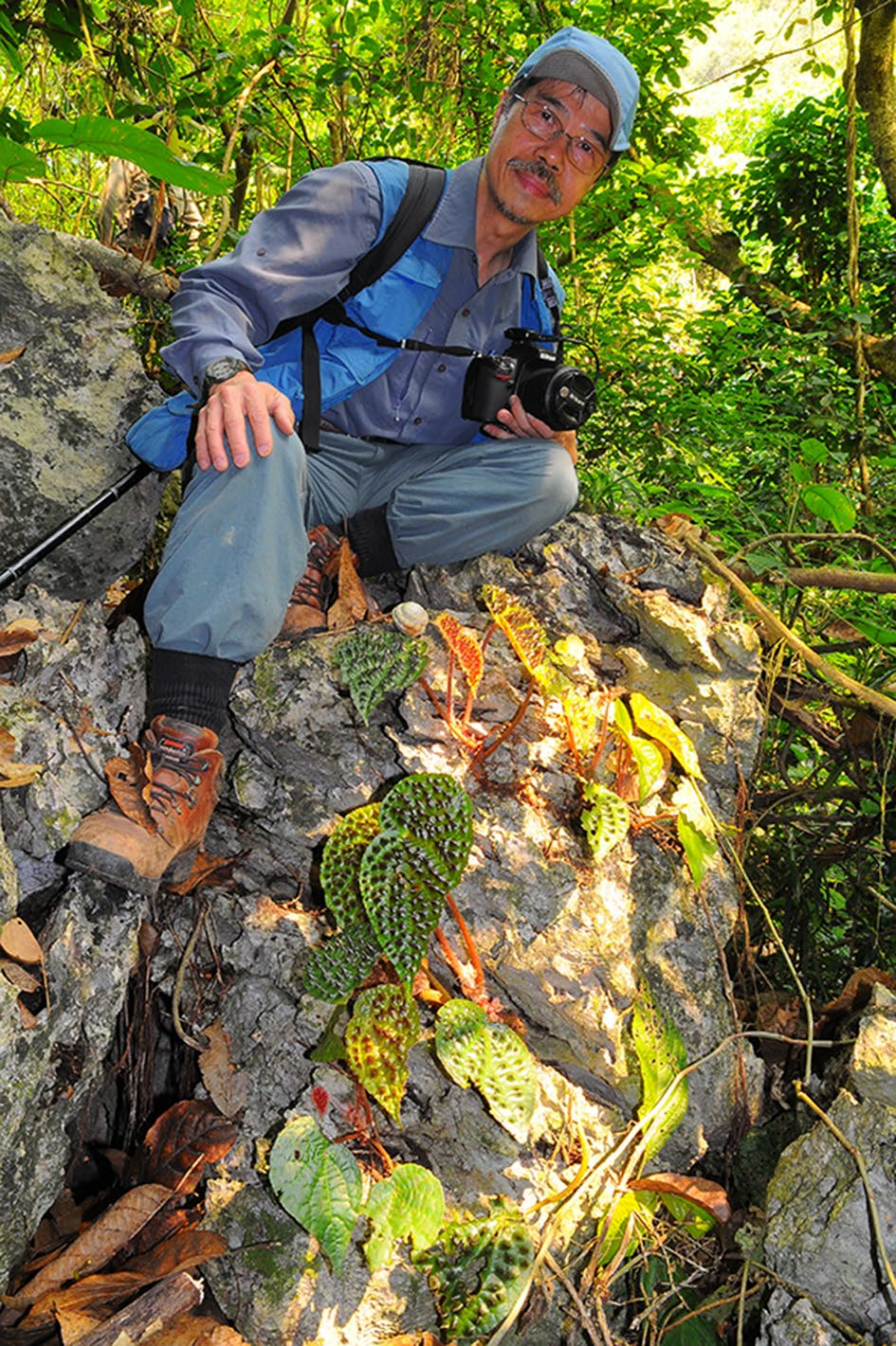
Ching-I Peng in the type locality of *Begonia ferox* C.I Peng & Yan Liu (Peng et al. 2013). Taken on April 20, 2011

**Table 1:** Eponymy

Begoniaceae
*Begonia chingipengii* Rubite in Rubite et al., Phytotaxa 164(3): 177, *figs. 1, 2* (2014)
*Begonia pengchingii* Phutthai & M.Hughes, Edinburgh J. Bot. 74(2): 153, 155, *fig. 3* (2017)
*Begonia pengii* S.M.Ku & Yan Liu in Ku et al., Bot. Stud. 49(2): 167, 170–171, *figs. 1, 2, 3A* (2008)
Berberidaceae
*Berberis pengii* C.C.Yu & K.F.Chung, Phytotaxa 184(2): 85, *figs. 7E–H, 13* (2014)
Cyperaceae
*Carex pengii* X.F.Jin & C.Z.Zheng, Taxon. Carex sect. Rhomboidales 222, *fig. 6-35* (2013)
Gesneriaceae
*Primulina pengii* W.B.Xu & K.F.Chung in Gu et al., Bot. Stud. 56(34): 6–8, *figs. 5, 6* (2015)
*Primulina chingipengii* W.B.Xu & K.F.Chung in Xu et al., Bot. Stud. 60(18): 13–14, *figs. 11, 12* (2015)
Onagraceae
*Epilobium pengii* C.J.Chen, Hoch & P.H.Raven, Syst. Bot. Monogr. 34: 169–170, *fig. 61* (1992)
Vitaceae
*Pseudocayratia pengiana* J.W.Hsu & J.Wen in Wen et al., J. Syst. Evol. 56(4): 378, *fig. 4* (2018)

## Ching-I Peng’s academic career

**Fig. 2:**
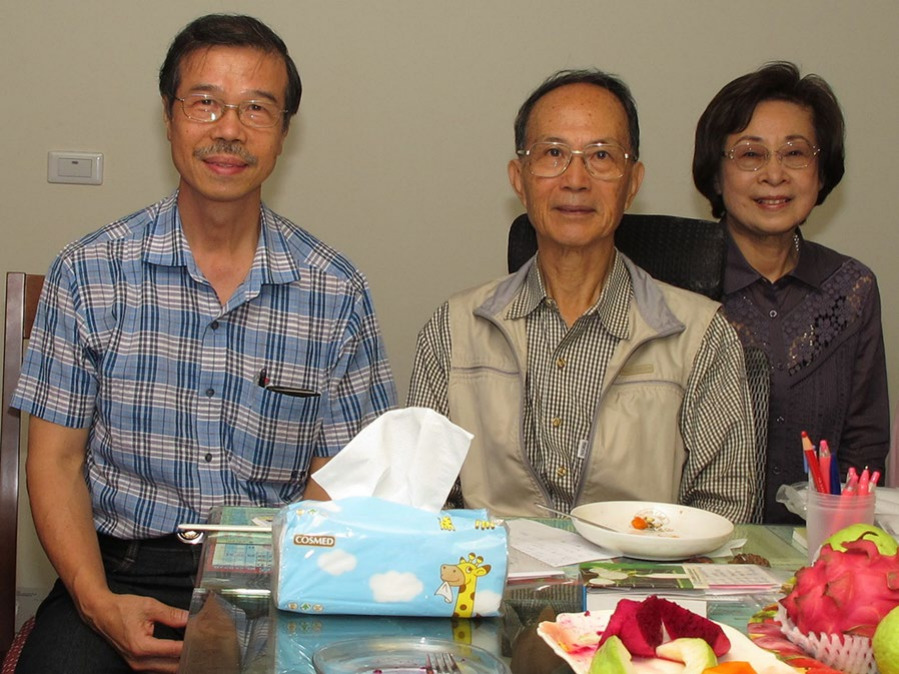
Ching-I Peng, Chien-Chang Hsu (許建昌; central), and Ms. Hsu (right). Taken on November 10, 2013

Ching-I Peng received his bachelor’s degree from the Department of Botany of National Chung Hsing University in 1972. After completing military service, he was admitted to the master’s program of the Research Institute of Botany of National Taiwan University in the fall of 1974. He received his master’s degree in the summer of 1976. Under the supervision of Dr. Chien-Chang Hsu (許建昌; Fig. 2), he studied the taxonomy and cytology of the Asteraceae of Taiwan. His master’s thesis “*Systematic Studies on Taiwan Compositae with a Chromosome Count*” (Peng 1978; Peng and Hsu 1978) and subsequent works established him as the leading expert on the family in Taiwan, evidenced by his treatments of Asteraceae in the Flora of Taiwan, 2nd edition (Peng et al. 1998) and the Manual of Taiwan Vascular Plants (Peng and Chung 2000). During his lifetime, he published 36 articles on Asteraceae (Additional file 2; A-1–A-36), the third most species-rich plant family in the Taiwanese flora, including 7 new taxa, 5 new names and 32 new distribution records (Additional files 3, 4).

**Fig. 3:**
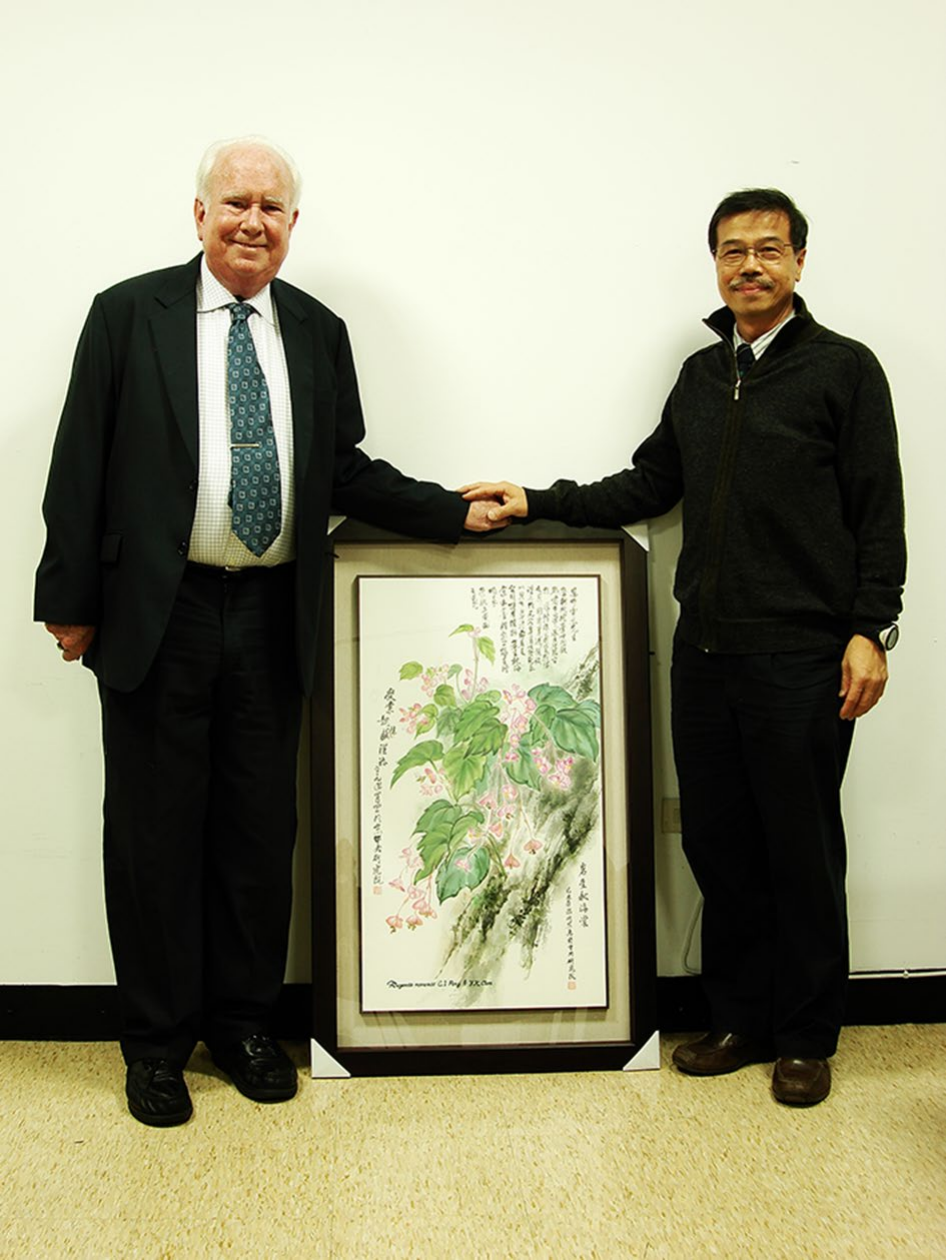
Ching-I Peng and Peter Raven with a water painting of *Begonia ravenii* drawn by Ming-Chao Yu (游明照). Taken in Academia Sinica, Taipei on April 7, 2009

**Fig. 4:**
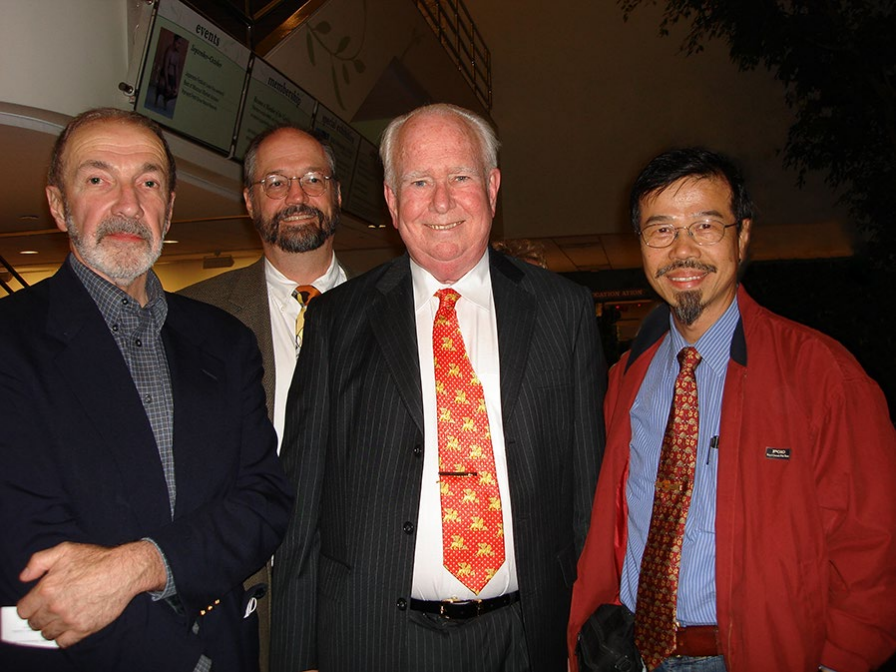
Ching-I Peng and his Missouri Botanical Garden connections. From left to right: David E. Boufford, Peter C. Hoch, Peter H. Raven, and Ching-I Peng. Taken during 53rd Annual Systematics Symposium, Missouri Botanical Garden “The Impact of Peter Raven on Evolutionary and Biodiversity Issues in the 20th and 21st Centuries” on October 14, 2006

After assistantships at the Yangmei Branch of the Taiwan Livestock Research Institute (1976–1977) and the Institute of Botany of Academia Sinica with Dr. Chang-Hung Chou (1978), Ching-I Peng started his PhD study in the summer of 1978 in the joint program of the Department of Biology at Washington University-St. Louis and the Missouri Botanical Garden, working under the supervision of Peter Raven. Ching-I Peng’s dissertation “*A Biosystematics Study of* Ludwigia *sect.* Microcarpium *(Onagraceae)*” is a classic in the field, resulting from tireless and meticulous cytogenetic analyses of thousands of *F*_*1*_ seedlings from artificial hybridizations among species of the section. His work greatly clarified the taxonomy of this notoriously complicated aquatic genus in the southeast USA (Peng 1989) and revealed a complicated history of hybridization and polyploidization (Peng 1988). Dr. Peng retained a life-time interest in *Ludwigia* and other taxa of Onagraceae, producing 18 publications on the family, covering taxonomy, biosystematics, chromosome cytology, anatomy, phytochemistry, conservation and molecular phylogenetics (Additional file 2; O-1–O-18). The latest is a molecular phylogenetic study (Liu et al. 2020) that tested and confirmed his hypotheses of evolutionary relationships in the north temperate haplostemonous *Ludwigia* drawn from cytological data (Peng 1988; Peng et al. 2005c). Throughout his career, Dr. Peng maintained a close connection with Peter Raven (Fig. 3) and the Missouri Botanical Garden (Fig. 4), which fostered him as one of the most influential contemporary East Asian taxonomists.

At the end of 1982, after a short postdoc at the Missouri Botanical Garden, Dr. Peng assumed the position of Associate Research Fellow in the Institute of Botany of Academia Sinica where he spent the rest of his professional career. In his new role, Dr. Peng devoted himself fully to better understand the systematics and evolution of the flora of Taiwan and East Asia (Peng 1992). With his assistants and students, he collected extensively not only in a wide range of natural habitats, but also in disturbed and previously neglected habitats such as graveyards and lowland foothills (Hsu 2018a). As an extremely patient and attentive collector, Dr. Peng trained his assistants and students to make high quality specimens and to record field details about topography, phenology, and morphology. During his lifetime, Dr. Peng collected and numbered 25,139 specimens often with several duplicates per number, making him perhaps the most prolific collector in Taiwan. With his dedication, Dr. Peng not only restored the function of the institute’s herbarium [registered to Index Herbariorum (Thiers 2015) as HAST (Herbarium, Institute of Botany, Academia Sinica, Taipei) by Ching-I Peng in 1982] but also established an extensive specimen exchange network with domestic (TAI, TAIE, TAIF, TNM, and TNU) and major herbaria around the world [Australia (CANB), China (IBSC, KUN, and PE), Japan (KANA, KYO, MAK, OOM, RYU, SAPA, TI, and TNS), Korea (SKK), Malaysia (UKMB and SAN), Netherlands (L), New Zealand (CHR), the Philippines (PNH), Singapore (SING), UK (BM and E), and USA (A, BRIT, CAS, DOV, MO, OSH US, and PTBG)]. Under Dr. Peng’s direction, the collection of HAST expanded from a few thousands in 1982 to more than 140,000 specimens today. Through his visionary leadership, HAST entered the digital era in 1995, first by establishing a searchable database of label information, and then by initiating the imaging of collections in 2002 with the support of the National Digitization Project of the National Science Council. Currently, HAST’s Database of Native Plants in Taiwan (https://hast.sinica.edu.tw/), hosting label information, specimen images, and photographs of plants in the field, are accessed by more than 5,000 visitors around the world each month (e.g., Keil and Chase 2019; Lin et al. 2019).

Dr. Peng was exceptionally fluent in English, which enabled him to establish numerous and diverse collaborations with foreign scholars. In addition to his work on Asteraceae and Onagraceae, his international collaborations facilitated his studies of *Amorphophallus* (Hetterscheid and Peng 1995), *Begonia* (see below), Brassicaceae (Yang and Peng 1996; Al-Shehbaz and Peng 2000; Hsu et al. 2005), Campanulaceae (Peng and Lammers 1998; Kokubugata et al. 2006a; Hsu et al. 2011a), Fabaceae (Hsing et al. 2001; Ho et al. 2011), Gesneriaceae (Kokubugata and Peng 2004; Kokubugata et al. 2011), Liliaceae *s.l.* (Kokubugata et al. 2004; Peng et al. 2007; Saito et al. 2009; Hsu et al. 2011b), Primulaceae (Peng and Hu 1999; Anderberg et al. 2001; Kokubugata et al. 2006b; Yan et al. 2007; Kokubugata et al. 2008; Yan et al. 2010; Kono et al. 2012b; Wanntorp et al. 2012), Ranunculaceae (Kita et al. 1997), and Rosaceae (Naruhashi et al. 1999, 2002; Hsu et al. 2007; Chang et al. 2011). With 281 publications (Additional file 2), Dr. Peng is the most productive Taiwanese plant taxonomist of his generation. He published 121 plant names new to science (Additional file 3) and added an additional 71 new records to the flora of Taiwan (Additional file 4).

Dr. Peng’s contributions extended far beyond basic research (Additional file 1). He was an adjunct professor at three universities (National Taiwan Marine College, National Taiwan Normal University, and National Cheng-Kung University), and trained numerous students and research assistants, many of whom now occupy important academic and governmental positions. He served as the Deputy Director (1995–1997) and then Adjunct Director (1996–1998) of the National Museum of Natural Science where he helped to establish the botanical garden and served as director (2003–2006). He was a member of numerous academic associations, serving in several important positions, including as a council member of the International Association of Plant Taxonomists (1999–2005) and the Taiwan Society of Plant Systematics (2006–2017). Before his retirement, Dr. Peng had been Editor-in-Chief, Co-Editor in Chief, and Associate editor of *Botanical Studies* and its predecessor *Botanical Bulletin of Academia Sinica*. He also served on the editorial boards of several important journals and floras, including as managing editor of Volume 4 of the Flora of Taiwan, 2nd edition (1996–1998) and editorial board member for the Flora of China (1997–2013). Throughout his career, Dr. Peng organized many domestic and international conferences and edited numerous highly influential conference proceedings, including the first Cross-strait Symposium on Floristic Diversity and Conservation in 1997 (Chiu and Peng 1998) and the International Symposium on the Future of Biodiversity in Taiwan in 2000, which led to the establishment of the Biodiversity Research Center of the Academia Sinica (BRCAS) in 2005. Because of his reputation as the leading plant taxonomist of Taiwan, Dr. Peng was invited as an advisory board member for important governmental organizations of conservation and education, including the Taiwan Endemic Species Research Institute (1992–2018), the Taiwan Forestry Research Institute (1996–2018), and the National Museum of Natural Science (1998–2018).

## Ching-I Peng’s pursuit of *Begonia*

With 1963 currently accepted species (Hughes et al. 2015a), the mega-diverse genus *Begonia* L. is perhaps the fifth largest flowering plant genus. During the last two decades, it has doubled in size from ca. 900 species in 1997 (Frodin 2004) to its current size, making it also the fastest growing genus of flowering plants (Moonlight et al. 2018). A major impetus of the phenomenal growth of *Begonia* during the past few decades has been the passion and dedication of Ching-I Peng and his collaborations with *Begonia* researchers and enthusiasts around the world, which resulted in 81 publications (Additional file 2; *B*-*1*–*B*-*81*) and 98 new species of *Begonia* (Additional file 3).

Dr. Peng’s interest in *Begonia* was ignited in 1985 by a fortuitous field trip guided by Hsin-Fu Yen (嚴新富) that resulted in the collection of a deciduous, tuberous and stoloniferous species new to Taiwan. The new species was named *B. ravenii* C.I Peng & Y.K.Chen to honor Peter Raven (Fig. 3) for his mentorship and contribution to plant systematics and evolution (Peng et al. 1988). Concurrently, Yung-Kuan Chen (陳永寬) revised the *Begonia* of Taiwan, resulting in the description of four additional novelties: *B. austrotaiwanensis* Y.K.Chen & C.I Peng (Peng and Chen 1990), *B. chuyunshanensis* C.I Peng an Y.K.Chen, *B. tengchiana* C.I Peng & Y.K.Chen, and *B. wutaiensis* C.I Peng & Y.K.Chen (Peng et al. 2005b). Through experimental hybridization and cytological studies, the enigmatic *B. buimontana* Y.Yamam. was shown to be a natural hybrid between *B. palmata* D.Don and *B. taiwaniana* Hayata (Peng and Chen 1991), and two new natural hybrids, *B. *× *taipeiensis* C.I Peng (Peng and Chiang 2000; Peng and Sue 2000; Chiang et al. 2001) and *B. *× *chungii* C.I Peng and S.M.Ku (Peng and Ku 2009; Kono et al. 2012a), were described. Largely through his efforts, the number of *Begonia* species known from Taiwan increased from 7 (Liu and Lai 1977) to 19 (Oginuma and Peng 2002; Peng et al. 2005b; Nakamura et al. 2015).

**Fig. 5:**
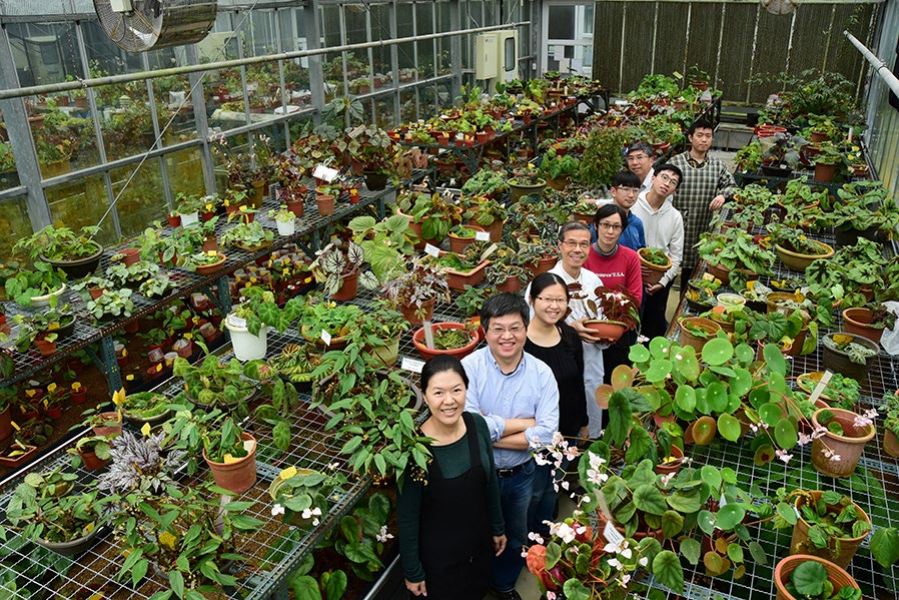
Ching-I Peng with BRCAS’s *Begonia* research team [from lower left to right: Yi-Shan Li (李宜珊; *Begonia* caretaker), Kuo-Fang Chung, Hsun-An Yang (楊巽安; research assistant), Ching-I Peng, Yu-Hsin Tseng (曾妤馨; postdoc), Wen-Hsi Kuo (郭聞喜; research assistant), Yu-Lan Huang (黃郁嵐; research assistant), Ku-Feng Lin (林谷峰; manager of the greenhouse core facilities), and Kuan-Pin Chen (陳觀斌; *Begonia* caretaker)] in the Experimental Greenhouse, Biodiversity Research Center, Academia Sinica collection. Photograph taken on March 21, 2017, approximately 3 weeks before Dr Peng was diagnosed with leukemia

Because most *Begonia* species are semi-succulent plants containing a high proportion of water, morphological characters such as coloration and variegation are very poorly preserved in the dried condition; thus species identification and description based on herbarium specimens alone is extremely difficult (Hughes and Girmansyah 2011). Consequently, Dr. Peng not only collected but also cultivated *Begonia* in the greenhouse to observe morphology and life history. Dr. Peng’s 30-year effort amassed a living collection of more than 500 wild *Begonia* species (Fig. 5) in the Experimental Greenhouse of BRCAS, with duplication in the Dr. Cecilia Koo Botanic Conservation Center (KBCC). The living *Begonia* collection in BRCAS and KBCC is one of the largest in the world (Hughes and Peng 2018).

In the summer of 1999, Dr. Peng began to extend his *Begonia* research to China where he first established a collaboration with Yu-Min Shui (稅玉民), then a PhD student supervised by Cheng-Yih Wu (吳征鎰) of the Kunming Institute of Botany, Chinese Academy of Science. In 2002, Shui, Wu, and Peng published “*Synopsis of the Chinese species of* Begonia *(Begoniaceae), with a reappraisal of sectional delimitation* (Shui et al. 2002),” providing a foundation for the taxonomic revision of Chinese *Begonia*. Thereafter, Raven, Co-chair of the editorial committee of the Flora of China (FOC) project, invited him to contribute to the treatment of Begoniaceae for the FOC.

To gain first-hand insight into the diversity of Chinese *Begonia* for the FOC, Dr. Peng’s team travelled to China 15 times, visiting herbaria and type localities, exploring Guangxi, Yunnan, Guangdong, Guizhou, and Hainan, and establishing further collaborations with Chinese botanists. By 2006, 17 new species and one new distribution record of Chinese *Begonia* were reported (Ku et al. 2004; Ye et al. 2004; Li et al. 2005; Liu et al. 2005; Peng et al. 2005a, b, c; Fang et al. 2006; Ku et al. 2006). The resulting treatment of Begoniaceae in the FOC (Gu et al. 2007) covered 173 species. The treatment constituted a full taxonomic revision of the Chinese *Begonia*, contrasting with the majority of previous treatments for FOC, which mainly translated and updated the Chinese *Flora Reipublicae Popularis Sinicae* (FRPS) into English.

**Fig. 6:**
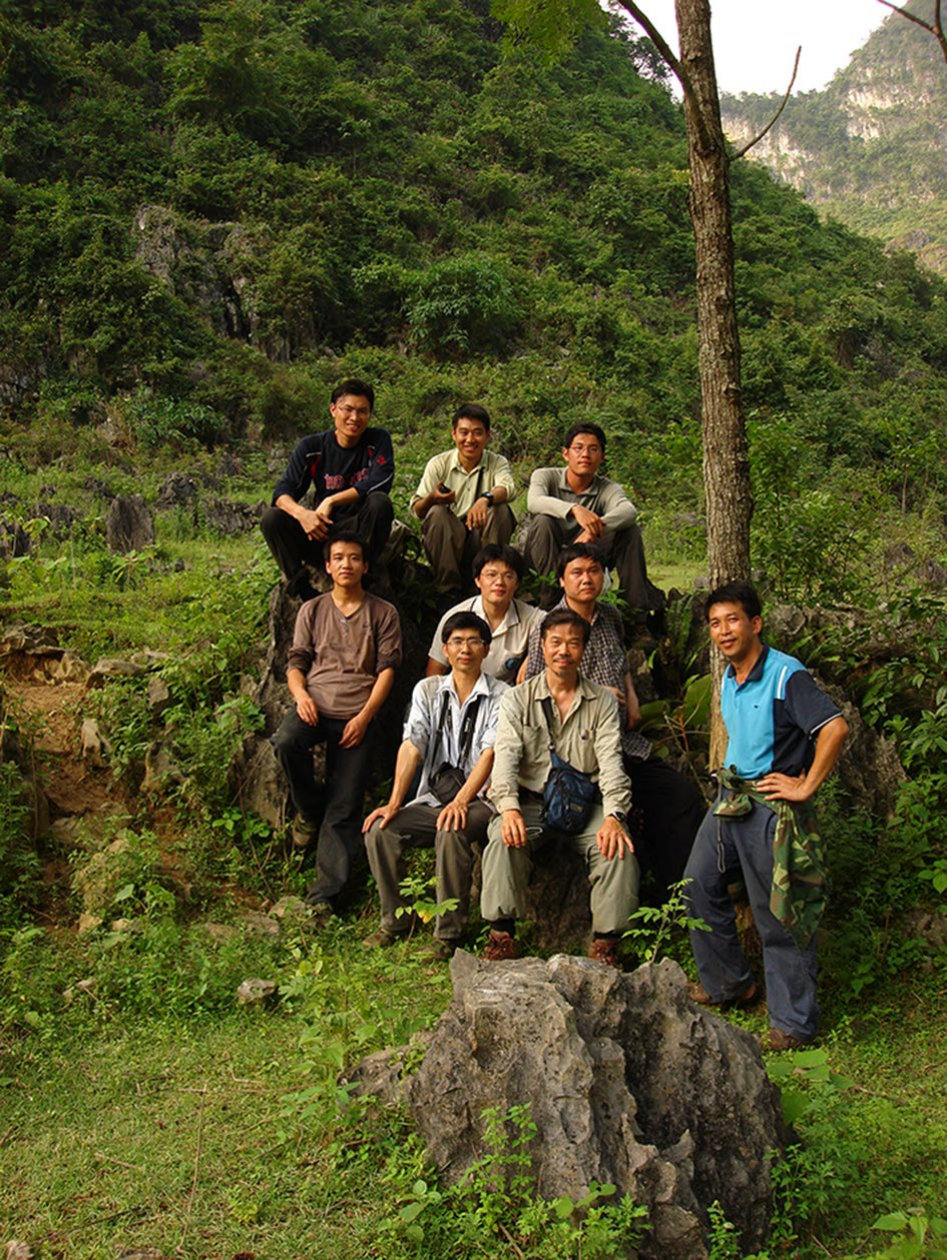
Field trip in Guangxi. Back row from left to right: Bo Pan (盘波; Guangxi Institute of Botany, IBK), Wei-Bin Xu (许为斌; IBK), and Chih-Kai Yang (楊智凱; BRCAS). Middle row: Gao-Zhong Pu (蒲高忠; IBK) Shin-Ming Ku (古訓銘; BRCAS), and Yun-Fei Deng (邓云飞; South China Botanical Garden). Front row: Yan Liu (刘演; IBK), Ching-I Peng, and Shi-Nian Lu (陆仕念). Taken on June 24, 2008

Dr. Peng visited Guangxi, China for the first time in 2002 and was immediately captivated by its splendid landscape of limestone karsts and the diverse and underexplored *Begonia* flora. He soon established a close collaboration with Professor Yan Liu (刘演; Fig. 6) of the Guangxi Institute of Botany, and visited Guangxi a total of 18 times. Together their collaboration resulted in some 30 published articles (Additional file 2), including the description of 17 new *Begonia* species (Dong and Liu 2019). In particular, their collaboration greatly improved knowledge of the taxonomy and evolution of *Begonia* sect. *Coelocentrum* Irmsch. (Liu et al. 2005; Peng et al. 2005a; Chung et al. 2014; Tseng et al. 2019), one of the most characteristic groups of limestone plants in Guangxi (Xu et al. 2019).

**Fig. 7:**
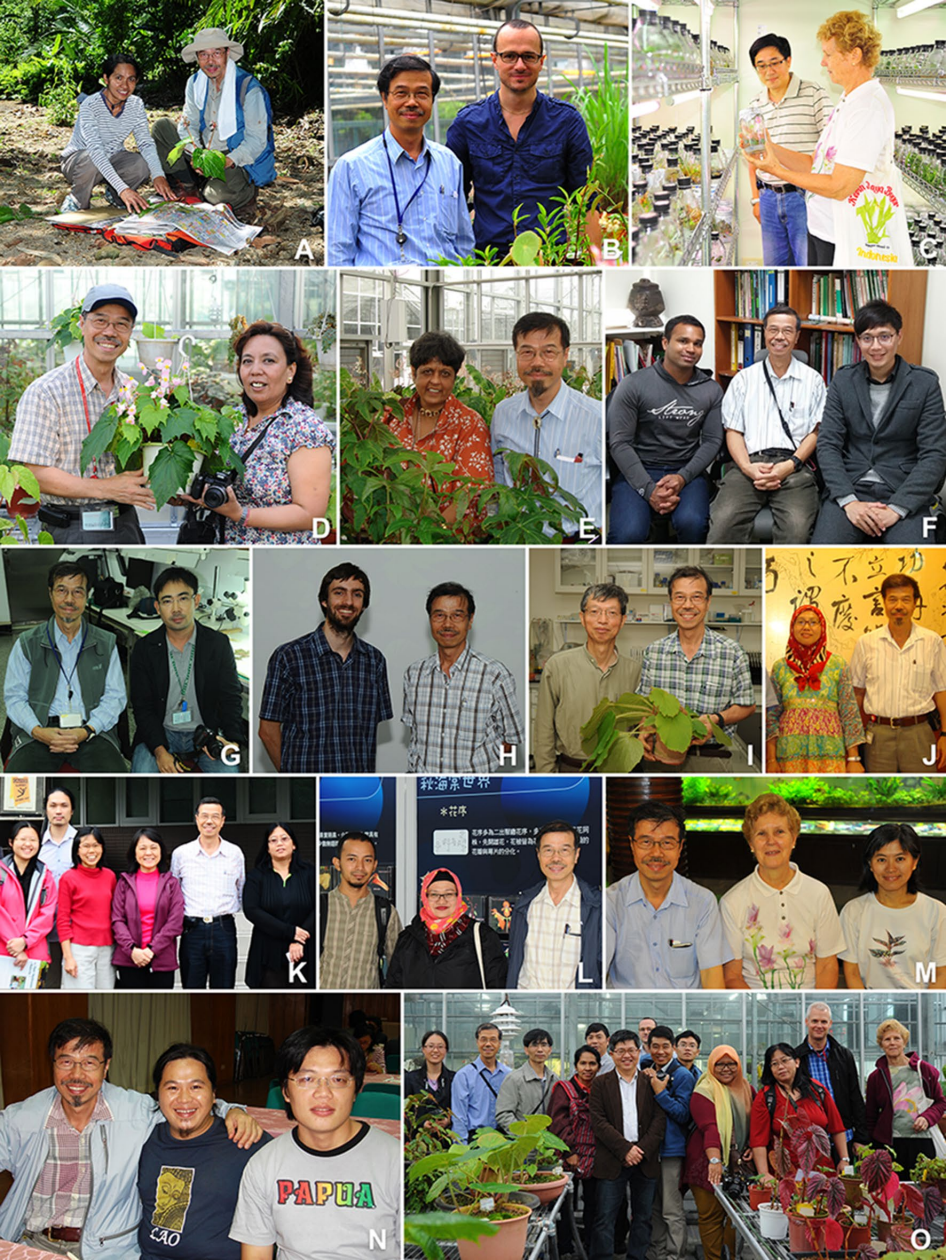
Ching-I Peng (CIP) and *Begonia* researchers and enthusiasts. **a** Rosario R. Rubite (University of the Philippines Manila; UP Manila) and CIP, June 21, 2012. **b** CIP & Mark Hughes (Royal Botanic Garden Edinburgh; RBGE), April 5, 2010. **c** Wei-Hsin Wu (National Museum of Natural Science, Taichung) & Ruth Kiew (Forest Research Institute, Malaysia; FRIM), June 16, 2010. **d** CIP and Sangeeta Rajbhandary (Tribhuvan University, Nepal), May 7, 2012. **e** Rekha Morris (South Carolina, USA) and CIP. **f** Dylan Cousin (The Melbourne Begonia Society, Australia), CIP, and Che-Wei Lin (林哲緯; Taiwan Forestry Research Institute), January 4, 2016. **g** CIP & Thamarat Phutthai (Mahidol University, Thailand), December 21, 2011. **h** Peter W. Moonlight (RBGE) & CIP, July 11, 2013. **i** Etsuo Kobayashi (小林悅夫; Japanese *Begonia* Society) & CIP with *Begonia togashii* (Tanaka and Peng 2016), June 18, 2015. **j** Rimi Repin (Sabah Park, Malaysia) & CIP, August 7, 2008. **k** Hsun-An Yang (楊巽安; BRCAS), Wai-Chao Leong (梁慧舟; BRCAS), Yoke Mui Chan (FRIM), Lucy Chong (Sarawak Forestry, Malaysia; SAR), CIP and Julia Sang (SAR), November 24, 2013. **l** Wisnu H. Ardi (Bogor Botanic Gardens, Indonesia; BO), Hartutiningsih-M. Siregar (BO), and CIP, December 10, 2013. **m** CIP, Ruth Kiew (FRIM), and Chiou-Rong Sheue (許秋容; National Chung Hsing University), June 16, 2010. **n** CIP, Hieu Quang Nguyen (Center for Plant Conservation of Vietnam), and Shin-Ming Kuo (古訓銘; BRCAS), October 28, 2008. **o** Lina Dong (Guangxi Institute of Botany, China; IBK), CIP, Yan Liu (IBK), Rosario Rubite (UP Manila), Bangping Cai (蔡邦平; Xiamen Botanical Garden), Kuo-Fang Chung (BRCAS), Mark Hughes (RBGE), Wei-Bin Xu (IBK), Yu-Song Huang (IBK), Rafidah Rahman (FRIM), Julia Sang (SAR), Daniel C. Thomas (Singapore Botanic Gardens), and Ruth Kiew (FRIM), April 12, 2015. **b**, **d**, **f**, **g**, **h**, **i**, **j**, **k**, **l**, **n**, and **o** were taken in Academia Sinica

Following the completion of the FOC treatment of Begoniaceae, Dr. Peng initiated field studies of *Begonia* in India (1 trip), Indonesia (2 trips), Malaysia (3 trips), the Philippines (5 trips), Thailand (3 trips), and Vietnam (3 trips), and visited public and private collections of *Begonia* in Australia, Denmark, France, India, Japan, Netherlands, UK, and the United States. BRCAS’s expanding living collection of *Begonia* became an invaluable asset for both research and conservation, especially with its many collections from type localities (Hughes and Peng 2018). Dr. Peng constantly received requests for literature, species identification, advice on *Begonia* cultivation, as well as plant material exchange. Gradually Academia Sinica became the hub of Asian *Begonia* research and conservation, hosting and welcoming both domestic and international *Begonia* researchers and enthusiasts from countries around the world, including Australia, China, India, Indonesia, Japan, Malaysia, Nepal, the Philippines, Singapore, Thailand, Vietnam and the UK (Fig. 7). Dr. Peng’s hospitality fostered fruitful collaborations, including detailed studies of *Begonia* of the Philippines with Rosario Rubite (Fig. 7a) of the University of the Philippines Manila and Mark Hughes (Fig. 7b) of the Royal Botanic Garden Edinburgh that led to the discovery of many new species (e.g., Peng et al. 2017; Hughes et al. 2018; Rubite et al. 2018), the recircumscription of *Begonia* sect. *Baryandra* (Rubite et al. 2013), phylogeographic (Nakamura et al. 2012, 2014) and biogeographic (Hughes et al. 2015a, b) studies of the section, and the detection of the first hybrid *Begonia* in the Philippines (Liu et al. 2019). The global network established through Dr. Peng’s dedication resulted in the publication of a total of 98 new species, including 29 species from China, two from Indonesia, three from Myanmar, 25 from Malaysia, 15 from the Philippines, ten from Taiwan, one from Thailand, and 13 from Vietnam (Additional file 3).

**Fig. 8:**
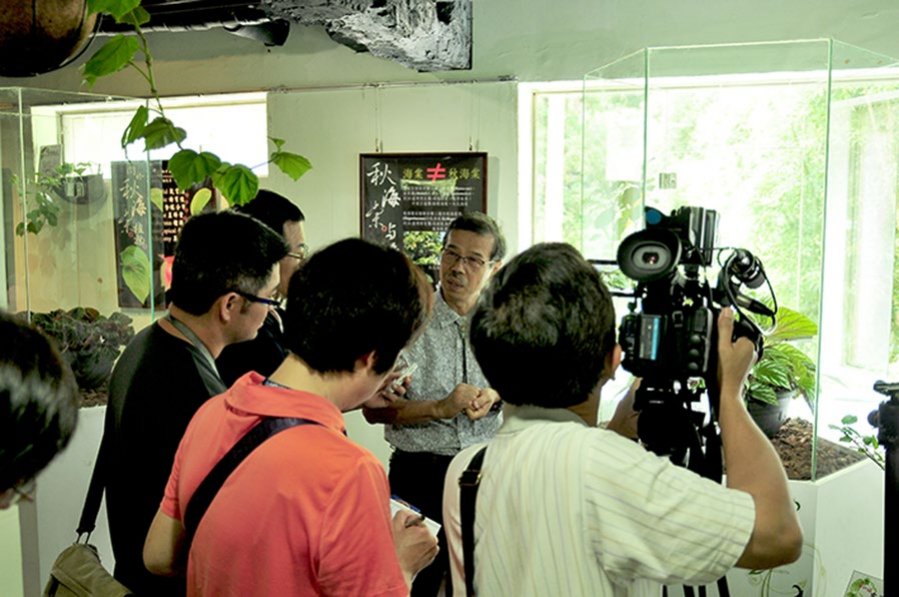
Ching-I Peng guided the audience to the *Begonia* exhibition in Xitou Nature Education Area, National Taiwan University Experimental Forest. Taken on July 1, 2016. Courtesy of Chih-Kai Yang

Dr. Peng also enthusiastically promoted the aesthetic beauty and the conservation of *Begonia*. During the periods of 2008–2011 and 2013–2015, Dr. Peng’s lab produced *Begonia* calendars and postcards for colleagues, friends, and collaborators that were extremely popular among *Begonia* enthusiasts. Dr. Peng was invited to give numerous public lectures both in Taiwan and abroad to share his knowledge of *Begonia* diversity (Fig. 8) and adventurous stories of collecting *Begonia* in exotic places, which included being hospitalized after being attacked by a swarm of bees in Sabah and breaking ribs in Guizhou. During the 2010 Taipei International Flora Exposition, BRCAS’s living collection of *Begonia* was featured in a special exhibition in the Pavilion of Future (2011.02.11–2011.04.25). More recently a subset of BRCAS’s *Begonia* living collection duplicated in the National Museum of Natural Science and curated by Dr. Wei-Hsin Hu (胡維新; Fig. 7c) was showcased in the Exhibition of Wild Begonias Breeding and Germplasm Conservation (2017.01.25–2017.04.23) in the Museum. A month after Dr. Peng’s passing, “*Asian Begonia: 300 Species Portraits*” co-edited by Hughes and Peng (2018) was published, reflecting his long-standing commitment to the study of *Begonia* and his lengthy and extensive collaborations with international scholars of Asian *Begonia*. Subsequently, Dr. Peng’s family edited the book “為愛走天涯:踏覓秋海棠 (*Endless Trekking in Search of Begonia*)” based on his lecture notes, travel logs, and photographs of his calendars to remember Dr. Peng’s ever-lasting passion for *Begonia*.

During his prolific academic career, Dr. Peng published 81 scientific publications on *Begonia* with more than 100 co-authors, including 35 research articles published in *Botanical Studies* and its predecessor *Botanical Bulletin of Academia Sinica* (Additional file 2). He and his collaborators described 98 taxa of *Begonia* new to science (Additional file 3), greatly improving our understanding of the diversity of Asian *Begonia*. His exquisite taxonomic works of *Begonia*, always featuring superb illustrations, high-quality colored photographs taken both in the field and studio, and chromosome cytology (e.g., Peng et al. 2017; Tseng et al. 2017; Hughes et al. 2018), have set a very high standard for the systematic study of Asian *Begonia*, contributing greatly to the resurgence and recent renaissance of *Begonia* discoveries (e.g., Pham et al. 2019; Wang et al. 2019). Utilizing BRCAS’s *Begonia* living collection, Dr. Peng and collaborators pioneered chromosome cytology (Peng and Chen 1991; Peng and Sue 2000; Oginuma and Peng 2002; Kono et al. 2012a) and in vitro cultivation (Hu et al. 2010) in *Begonia* and conducted novel experiments to understand the nature of leaf variegation (Sheue et al. 2012) and iridescence in the genus (Pao et al. 2018). Integrating molecular data in their taxonomic studies, Dr. Peng’s team also contributed significantly to a more natural infrageneric classification of *Begonia* (Rubite et al. 2013; Chung et al. 2014; Moonlight et al. 2018). Such international collaborations have also greatly advanced understanding of the biogeographic histories and evolutionary mechanisms that underlie the massive diversification of *Begonia* on both regional (Chung et al. 2014; Nakamura et al. 2014; Hughes et al. 2015; Hughes et al. 2018; Tseng et al. 2019) and continental scales (Moonlight et al. 2015). His research is also the most important source of critical baseline data for the conservation of many critically endangered *Begonia* species (Sang and Kiew 2014), the majority of which are threatened by habitat destruction and overexploitation (Clements et al. 2006).

**Fig. 9:**
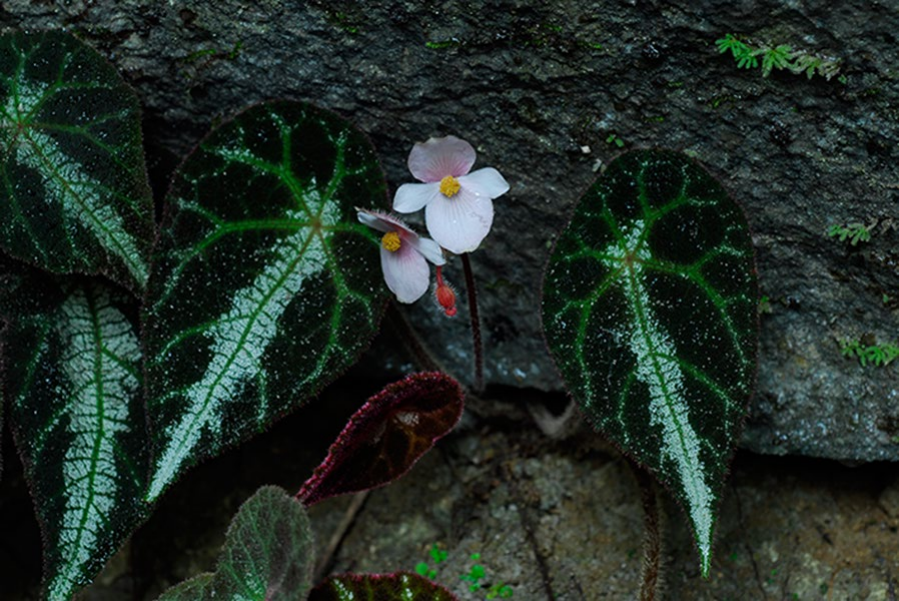
*Begonia pengii* S.M.Ku & Yan Liu (Ku et al. 2008) in the type locality

**Fig. 10:**
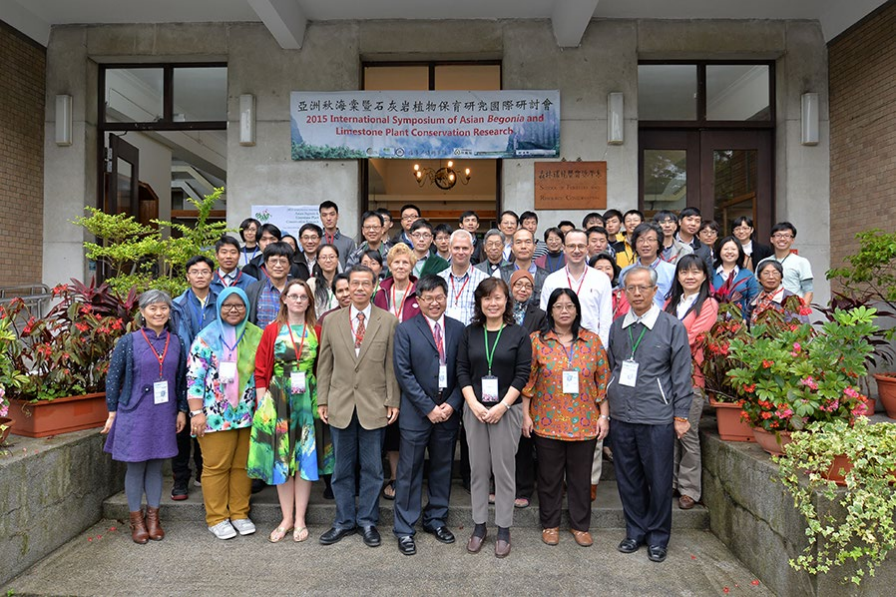
Participants of the 2015 International Symposium of Asian *Begonia* and Limestone Plant Conservation Research. Taken on April 11, 2015

To honor his remarkable achievements, a total of 9 plant species were named after Ching-I Peng (Table 1), including *Begonia pengii* S.M.Ku and Yan Liu (Ku et al. 2008; Fig. 9), *B. chingipengii* Rubite (Rubite et al. 2014), and *B. pengchingii* Phutthai and M.Hughes (Phuttai and Hughes 2017). Toward his retirement, Dr. Peng’s outstanding academic career was recognized by the 2009 Outstanding Alumnus Award of the Department of Life Sciences, National Chung Hsing University and the 2016 Lifetime Achievement Award of the Taiwan Society of Plant Systematics (TSPS). On April 11–12, 2015, the International Symposium of Asian *Begonia* and Limestone Plant Conservation was organized to celebrate his retirement (Fig. 10).

Dr. Peng’s official retirement in August of 2015 released him from administrative duties and enabled him to focus fully on the taxonomy of *Begonia*. Dr. Peng co-authored 25 journal articles and described 29 new *Begonia* after his retirement. The majority of the manuscripts were prepared while he underwent chemotherapy after being diagnosed with leukemia in April of 2017. After his sudden passing, he was commemorated by the 2018 Lifetime Achievement Award of the Taiwan Society of Plant Biologists, the 2018 Eva Kenworthy Gray Award of The American Begonia Society, and 2019 Outstanding Alumnus Award of the College of Life Sciences, National Chung Hsing University. A special issue in *Nature Conservation Quarterly*, the official magazine of the Taiwan Endemic Species Research Institute, was published in autumn of 2018 to pay tribute to Dr. Peng (Chou 2018; Chung 2018; Hsu 2018a, b). In the spring of 2019, the TSPS’s annual conference was named Dr. Ching-I Peng Memorial Symposium of East Asian Plant Systematics. The symposium included a special section focusing on *Begonia* research. In addition to the invited talks that commemorated his contributions, the first Dr. Ching-I Peng’s Memorial Awards (彭鏡毅博士紀念獎), created and supported by the National Museum of Natural Science (NMNS), The NMNS Foundation, and TSPS, were awarded to encourage and motivate taxonomic studies in Taiwan.

To further commemorate Dr. Ching-I Peng’s botanical legacy, the Research Museum of BRCAS is collaborating with the Academia Sinica Center for Digital Cultures to launch a series of on-line exhibitions on the platform of Open Museum (https://brmas.openmuseum.tw/), including three exhibitions highlighting Dr. Peng’s work in the flora of Taiwan, *Ludwigia*, and Asteraceae. An exhibition featuring Dr. Peng’s *Begonia* research will be released by the end of 2020.

Beyond his considerable academic, research, and professional legacy, Ching-I Peng is remembered by his family, friends, colleagues and students as a kind, generous, positive, and thoughtful person with an engaging sense of humor. He had many interests and hobbies outside of botany, including collecting crafts and antiques of turtles, attending performances of Chinese opera, and playing Ping-Pong at noon-time with his former colleagues of the Institute of Botany. To this day, his personal Facebook page (https://www.facebook.com/profile.php?id=1278893812) is still constantly visited and posted by his family and friends around the world.

A week before he was transferred to the intensive care unit, I went to visit Dr. Peng in the hospital to discuss unfinished projects with him, not realizing that he would soon become too ill to speak. During my visit, he seemed perfectly fine, telling me that he has been blessed to have a great life surrounded by family and friends who always supported his pursuit of the passions that he truly loved. He had no regrets in his life. For those of us who know him well, his absence has been difficult to accept, but we take solace in many lasting accomplishments that he left behind. When I look back on the many years that I worked with him first as a research assistant, then as a postdoc, and finally as a professional colleague, I feel a great sense of gratitude and continual wonderment at his capacity to make the most of his life, and to always help others while doing it. While his remarkable scientific legacy lives on through his students, publications, and many other lasting accomplishments, his presence in our lives is sorely missed by us all.

## Supplementary information

**Additional file 1.** Ching-I Peng’s CV.

**Additional file 2.** Ching-I Peng’s bibliography.

**Additional file 3.** New taxa and names (121) published by Ching-I Peng.

**Additional file 4.** Ching-I Peng’s contribution to the flora of Taiwan: new distribution records (71 species).

## Data Availability

Not applicable.
