# Supplementary material for: In memoriam Ching-I Peng (1950–2018)—an outstanding scientist and mentor with a remarkable legacy
Source: Bot Stud. 2020 Apr 25;61:14. doi: 10.1186/s40529-020-00291-5 (PMC7182648; doi:10.1186/s40529-020-00291-5)
Supplement: Supplementary file 1 — Additional file 1. Ching-I Peng’s CV. [file 40529_2020_291_MOESM1_ESM.pdf]

Additional file 1. Ching-I Peng's CV.

Education:

B.S., Botany Department, National Chung Hsing University, Taichung, Taiwan, 1972

M.S., Botany Department, National Taiwan University, Taipei, Taiwan, 1976

Ph.D., Biology Department, Washington University, St. Louis, Missouri, USA, 1982

Appointments and Experience:

Research Fellow, Research Center for Biodiversity, Academia Sinica, Taipei, 2005.01–2015.07

Executive Director, Thematic Center for Systematics and Biodiversity Informatics, Research Center for Biodiversity, Academia Sinica, Taipei, 2004.01–2015.07

Research Fellow, Institute of Botany, Academia Sinica, Taipei, 1987.08–2004.12

Curator of Herbarium (HAST), Academia Sinica, Taipei, 1982–1995; 1998–1999; 2003–2004.

Associate Research Fellow and Herbarium Curator, Institute of Botany, Academia Sinica, Taipei, 1982.12–1987.07

Research Associate, Missouri Botanical Garden, 1982.05–1982.12

Research Assistant, Institute of Botany, Academia Sinica, Taipei, 1978.01–1978.07

Research Assistant, Yangmei Branch, Taiwan Livestock Research Institute, 1976.07–1977.12

Adjunct Associate Professor, National Taiwan Marine College, 1983–1984

Adjunct Associate Professor, National Taiwan Normal University, 1986–1987

Adjunct Professor, National Taiwan Normal University, 1987–1989

Research Fellow and Academic Deputy Director, National Museum of Natural Science, Taichung, Taiwan, 1995.12–1997.12

Acting Director, National Museum of Natural Science, Taichung, Taiwan, 1996.09–1997.10

Director, Botanical Garden, National Museum of Natural Science, Taichung, Taiwan, 2003-2006

Adjunct Professor, National Cheng-Kung University, 2002–2016

Society affiliations:

Taiwan Society of Plant Systematics, 2006–2018

Botanical Society of the Republic of China and Taiwan Society of Plant Biologist, 1983–2018

International Association for Plant Taxonomy, 1985–2018

American Society of Plant Taxonomists, 1979–2018

Editorial:

Co-Editor in Chief, Botanical Studies, 2007-2016

Associate editor, Botanical Studies, 2006

Associate editor, Botanical Bulletin of Academia Sinica, 1996 –2005

Editor-in-Chief, Botanical Bulletin of Academia Sinica, 1992–1995.

Editor, Index to Plant Chromosome Numbers, 1984–2016

Editor, Acta Phytotaxonomica Geobotanica (Japan), 1994–2018

Executive Editor, vol. 4, Flora of Taiwan, 2nd edition, 1996–1998

Editor, vol. 6, Flora of Taiwan, 2nd edition, 2002–2003

Advisory editorial board, Taiwan Journal of Forest Science, 1996–2018

Editorial consultant, Environmental Education Quarterly (Taiwan), 1996–2018

Editorial board, Flora of China, 1997–2013
Associate Editor, Chromosome Science (Japan), 1999–2018
Advisory board, Endemic Species Research, 2000–2018
Editorial Board, Guihaia (Guangxi, China), 2001–2018
Editorial board, Acta Phytotaxonomica Sinica (Beijing, China), 2004–2007
Editorial board, Journal of Systematics and Evolution (Beijing, China), 2008–2018
Editorial board, Korean Journal of Plant Taxonomy, 2009–2018
Editorial board, Bulletin of National Museum of Nature and Science, Series B (Botany) (Japan), 2010–2018

Committees/Councils:

Council member, GBIF ROC, 2003–2016
Council member, International Association for Plant Taxonomy, 1999–2005
Council member (1991–) and Executive Secretary, ROC Committee of the Pacific Science Association, 1991–1995
Council member (1992–) and Executive Secretary, ROC Committee of the International Union of Biological Sciences, 1992–1995
Council member, Botanical Society of the Republic of China, 1985–2016
Council member, Society of Wildlife and Nature ROC, 1997–2018
Council member, Biological Society of China, 1999–2018
Council member, Taiwan Society of Plant Systematics, 2006-2017
Advisory board, Taiwan Forestry Research Institute, 1996–2018
Advisory board, National Museum of Natural Science, 1998–2016
Advisory board, Taiwan Endemic Species Research Institute, 1992–2018
Advisory board, Committee of Life Sciences Terminology, National Institute for Compilation and Translation, 2007–2016
Advisory board, 2010 Taipei International Flora Exposition, 2009–2011

Scientific Symposia Organizer:

Symposium on Plant Resources and Landscape Conservation, Taipei, 1987
Workshop on the Biological Resources and Information Management of Taiwan, 1991
ROC-USA Symposium on Phytogeography and Botanical Inventory of Taiwan, 1992
International Symposium on Biodiversity and Terrestrial Ecosystems, Taipei, 1994
International Symposium on Rare, Threatened, and Endangered Floras of Asia and the Pacific Rim, Taipei, 1996
Workshop on Application of Information Systems on Botanical Inventory, Taipei, 1996
Workshop on the Botanical Inventory of Taiwan (I), Taipei, 1996,
Workshop on the Botanical Inventory of Taiwan (II), Taichung, 1997
Cross-strait Symposium on Floristic Diversity and Conservation, Taichung, 1997
Year 2000: Cross-strait Symposium on Biodiversity and Conservation, 2000
International Symposium on the Future of Biodiversity in Taiwan, Taipei, 2000

International Symposium on Plant Biodiversity and Development of Bioactive Natural Products, Taichung, 2001

International Symposium: Frontiers in Plant Science, Taipei, 2002

2003 International Symposium on Plant Diversity in Eastern Asia and Workshop on Botanical Gardens, Taichung, 2003

2004 International Symposium on Plant Diversity, Taipei, 2004

2015 International Symposium of Asian *Begonia* and Limestone Plant Conservation Research, Taipei, 2015

Awards:

2009 Outstanding Alumnus Award of the Department of Life Sciences, National Chung Hsing University

2016 Lifetime Achievement Award of the Taiwan Society of Plant Systematics

2018 Lifetime Achievement Award of the Taiwan Society of Plant Biologists

2018 Eva Kenworthy Gray Award of the American Begonia Society

2019 Outstanding Alumnus Award of the College of Life Sciences, National Chung Hsing University
