# Supplementary material for: In memoriam Ching-I Peng (1950–2018)—an outstanding scientist and mentor with a remarkable legacy
Source: Bot Stud. 2020 Apr 25;61:14. doi: 10.1186/s40529-020-00291-5 (PMC7182648; doi:10.1186/s40529-020-00291-5)
Supplement: Supplementary file 2 — Additional file 2. Ching-I Peng’s bibliography. [file 40529_2020_291_MOESM2_ESM.pdf]

Additional file 2. Ching-I Peng's bibliography. (*Chinese title translated into English by the author). For Peng's work of Asteraceae (A-), Onagraceae (O-), and *Begonia* (*B*-), the number within the parentheses denote chronical count of the publication.

1. **彭鏡毅**. (1976) *台灣菊科植物的系統分類與染色體數之研究*. 國立台灣大學植物研究所碩士論文, 台北市, 236 pp. [**Peng, C.-I** (1976) Systematic Studies on Taiwan Compositae with a chromosome count. Master Thesis, National Taiwan University, Taipei, 236 pp.]
2. 許建昌 & **彭鏡毅**. (1976) *台灣芻料植物圖譜*. 台灣省畜產試驗所楊梅分所刊物, 桃園縣, 84 pp. [Hsu, C.-C. & **Peng, C.-I** (eds.) (1976) Taiwan Forage Plants. Forage Department, Yangmei Branch, Taiwan Livestock Research Institute, Taoyuan, 84 pp.]
3. **Peng, C.-I** & Hsu, C.-C. (1978) Chromosome numbers in Taiwan Compositae. *Botanical Bulletin of Academia Sinica* 19: 53–66. (A-1)
4. **Peng, C.-I** (1978) Some new records for the flora of Taiwan. *Botanical Bulletin of Academia Sinica* 19: 83–86. (A-2)
5. **Peng, C.-I** (1982) A Biosystematic Studies of *Ludwigia* sect. *Microcarpium* (Onagraceae). PhD, Washington University, St. Louis.
6. **Peng, C.-I** & Kenton, A. (1982) Chromosome number of *Byblis liniflora* Salisb (Byblidaceae). *Annals of the Missouri Botanical Garden* 69: 414–415.
7. **Peng, C.-I** (1983) Triploidy in *Ludwigia* in Taiwan, and the discovery of *Ludwigia adscendens* (Onagraceae). *Botanical Bulletin of Academia Sinica* 24: 129–134. (O-1)
8. **Peng, C.-I**, Goldblatt, P. & Krukoff, B. A. (1983) Confirmation of the chromosome number in Cephalotaceae and Roridulaceae. *Annals of the Missouri Botanical Garden* 70: 197–198.
9. 彭鏡毅. (1984) 二倍體黃花水龍，妳在何方？. *中華植物學會通訊* 12: 6–7. [**Peng, C.-I** (1984) Diploid yellow-flowered primrose-willow, where are you? Bot. Soc. ROC Newsletter 12: 6–7.] (O-2)
10. **Peng, C.-I** (1984) *Ludwigia ravenii* (Onagraceae), a new species from the coastal plain of the southeastern United States. *Systematic Botany* 9: 129–132. (O-3)
11. **Peng, C.-I** & Kao, M.-T. (1984) *Calyptocarpus vialis* Less. (Asteraceae), a newly naturalized weed in Taiwan. *Botanical Bulletin of Academia Sinica* 25: 171–176. (A-3)
12. **Peng, C.-I** & Chen, Y.-F. (1985) *Hybanthus* Jacq (Violaceae), a new generic record for the flora of Taiwan. *Botanical Bulletin of Academia Sinica* 26: 213–220.
13. Huang, S. & **Peng, C.-I** (1985) Flavonoids of *Oenothera* (Onagraceae) in Taiwan. *Biological Bulletin of National Taiwan Normal University* 20: 87–91. (O-4)
14. 彭鏡毅 & 周昌弘. (1986) 玉山懸鉤子. *中華植物學會通訊* 15: 7–8. [**Peng, C.-I** & Chou, C.-H. (1986) *Rubus calycinoides*. Bot. Soc. ROC Newsletter 15: 7–8.]
15. **Peng, C.-I** (1986) A new combination in *Ludwigia* sect *Microcarpium* (Onagraceae). *Annals of the Missouri Botanical Garden* 73: 490–490. (O-5)
16. Chaw, S.-M., **Peng, C.-I** & Kao, M.-T. (1986) *Verbena bonariensis* L. (Verbenaceae): a newly naturalized plant in Taiwan. *Journal of Taiwan Museum* 39: 123–126.
17. **Peng, C.-I** & Huang, S. (1986) *Oenothera laciniata* Hill (Onagraceae), a previously

unrecorded species in Taiwan. *Botanical Bulletin of Academia Sinica* 27: 45–51. (O-6)

18. **Peng, C.-I**, Yen, S.-F. & Guo, J.-Y. (1986) Notes on the chromosome cytology of some rare, threatened, or endangered plants of Taiwan (I). *Botanical Bulletin of Academia Sinica* 27: 219–235. (A-3)
19. **Peng, C.-I** & Chaw, S. M. (1986) *Alysicarpus rugosus* (Willd) DC., a newly naturalized legume species in Taiwan. *Botanical Bulletin of Academia Sinica* 27: 247–253.
20. Chaw, S.-M. & **Peng, C.-I** (1987) Palynological notes on *Bretschneidera sinensis* Hemsl. *Botanical Bulletin of Academia Sinica* 28: 55–60.
21. Chou, C.-H., Hwang, S.-Y., **Peng, C.-I**, Wang, Y.-C., Hsu, F.-H. & Chung, N.-J. (1987) The selective allelopathic interaction of a pasture forest intercropping in Taiwan. *Plant and Soil* 98: 31–41.
22. **Peng, C.-I** (1987) *Murdannia spirata* (L.) Brückner (Commelinaceae), a neglected species in the Flora of Taiwan. *Journal of Taiwan Museum* 40: 51–56.
23. Chaw, S.-M. & **Peng, C.-I** (1987) Remarks on the species of Spermacoceae (Rubiaceae) of Taiwan. *Journal of Taiwan Museum* 40(1): 71–83.
24. 彭鏡毅. (1987) 從演化的觀點論高等植物種的觀念與天然雜交現象. *科學發展月刊* 15: 744–759. [**Peng, C.-I** (1987) An evolutionary perspective of the species concept of higher plants and natural hybridization*. *National Science Council Monthly* 15: 544–759.]
25. Huang, S., **Peng, C.-I** & Lee, H.-C. (1987) Flavonoid analyses of *Pyrola* (Pyrolaceae) in Taiwan. *Botanical Bulletin of Academia Sinica* 28: 283–287.
26. **Peng, C.-I** & Tobe, H. (1987) Capsule wall anatomy in relation to capsular dehiscence in *Ludwigia* sect. *Microcarpium* (Onagraceae). *American Journal of Botany* 74: 1102–1110. (O-7)
27. Chaw, S.-M. & **Peng, C.-I** (1987) *Spermacoce pusilla* Wall. (Rubiaceae) in Taiwan. *Journal of Taiwan Museum* 40(2): 57–59.
28. 周昌弘, 彭鏡毅 & 趙淑妙 (eds.) (1987) *台灣植物資源與保育*. 中華民國自然生態保育協會, 台北市, 238 pp. [Chou, C.-H., **Peng, C.-I** & Chaw, S.–M. (eds.) (1987) *Plant Resources and Conservation in Taiwan.* Society of Wildlife and Nature, ROC, 238 pp.]
29. **Peng, C.-I**, Chen, Y.-K. & Yen, H.-F. (1988) *Begonia ravenii* (Begoniaceae), a new species from Taiwan. *Botanical Bulletin of Academia Sinica* 29: 217–222. (*B-1*)
30. **Peng, C.-I** (1988) The biosystematics of *Ludwigia* sect. *Microcarpium* (Onagraceae). *Annals of the Missouri Botanical Garden* 75: 970–1009. (O-8)
31. Murata, G. & **Peng, C.-I** (1988) Newly naturalized North American plants in Midorogaike Lake, Kyoto. *Acta Phytotaxonomica et Geobotanica* 39: 150.
32. **Peng, C.-I**, Hu, L.-A. & Kao, M.-T. (1988) Unwelcome naturalization of *Parthenium hysterophorus* (Asteraceae) in Taiwan. *Journal of Taiwan Museum* 41: 95–101. (A-4)
33. Tobe, H., Raven, P. H. & **Peng, C.-I** (1988) Seed coat anatomy and relationships of *Ludwigia* sects *Microcarpium*, *Dantia*, and *Miquelia* (Onagraceae), and notes on fossil seeds of *Ludwigia* from Europe. *Botanical Gazette* 149: 450–457. (O-9)
34. **Peng, C.-I** (1989) The systematics and evolution of *Ludwigia* sect *Microcarpium*

(Onagraceae). *Annals of the Missouri Botanical Garden* 76: 221–302. (O-10)

35. Tobe, H. & **Peng, C.-I** (1990) The embryology and taxonomic relationships of *Bretschneidera* (Bretschneideraceae). *Botanical Journal of the Linnean Society* 103: 139–152.

36. **Peng, C.-I** (1990) *Ludwigia* × *taiwanensis* (Onagraceae), a new species from Taiwan, and its origin. *Botanical Bulletin of Academia Sinica* 31: 343–349. (O-11)

37. **Peng, C.-I** & Chen, Y.-K. (1990) *Begonia austrotaiwanensis* (Begoniaceae), a new species from southern Taiwan. *Journal of the Arnold Arboretum* 71: 567–574. (*B-2*)

38. Zardini, E. M., **Peng, C.-I** & Hoch, P. C. (1991) Chromosome numbers in *Ludwigia* sect. *Oligospermum* and sect. *Oocarpon* (Onagraceae). *Taxon* 40: 221–230. (O-12)

39. **Peng, C.-I** & Chen, Y.-K. (1991) Hybridity and parentage of *Begonia buimontana* Yamamoto (Begoniaceae) from Taiwan. *Annals of the Missouri Botanical Garden* 78: 995–1001. (*B-3*)

40. 彭鏡毅 (ed.) (1992) *台灣生物資源研究現況.* 中央研究院植物研究所專刊第11號, 台北市, 349 pp. [**Peng, C.-I** (ed.) (1992) *The Biological Resources of Taiwan: A Status Report*. Institute of Botany, Academia Sinica, Taipei, 349 pp.]

41. 彭鏡毅譯 (Peter H. Raven 撰). (1992) 建立國家生物資源資料庫的重要性. *In*: 彭鏡毅 (ed.) *台灣生物資源研究現況.* 中央研究院植物所專刊第11號, 台北市, pp. 1–12. [Raven, P. H. (1992) The importance of national biological inventory. *In*: **Peng, C.-I** [ed.], *The Biological Resource of Taiwan: A Status Report*, Institute of Botany, Academia Sinica, Taipei, pp. 1–12. Translated into Chinese by **Peng, C.-I**]

42. 彭鏡毅 & 楊遠波. (1992) 台灣種子植物之研究現況. *In*: 彭鏡毅 (ed.) *台灣生物資源研究現況.* 中央研究院植物研究所專刊第11號, 台北市, pp. 55–85. [**Peng, C.-I** & Yang, Y.-P. (1992) Status and research of the seed plant of Taiwan. *In*: **Peng, C.-I** [ed.], *The Biological Resources of Taiwan: A Status Report*, Institute of Botany, Academia Sinica, Taipei, 55–85.]

43. **Peng, C.-I** (ed.) (1992) Phytogeography and Botanical Inventory of Taiwan. Institute of Botany, Academia Sinica, Taipei, 91 pp.

44. Boufford, D. E. & **Peng, C.-I** (1993) *Soliva* Ruiz and Pavon (Anthemideae, Asteraceae) in Taiwan. *Botanical Bulletin of Academia Sinica* 34: 347–352. (A-5)

45. Raven, P. H. & **Peng, C.-I** (1993) Onagraceae. *In*: Editorial Committee of the Flora of Taiwan, n.e. (ed.) *Flora of Taiwan, 2nd edn. Vol. 3*. Editorial Committee of the Flora of Taiwan, 2nd edn., Taipei, pp. 937–967. (O-13)

46. Yang, S.-Z. & **Peng, C.-I** (1994) *Gardneria* (Loganiaceae) in Taiwan. *Botanical Bulletin of Academia Sinica* 35: 223–227.

47. **Peng, C.-I** & Chou, C.-H. (eds.) (1994) *Biodiversity and Terrestrial Ecosystems*. Institute of Botany, Academia Sinica, Taipei, 527 pp.

48. **Peng, C.-I**, Kuo, C.-M. & Yang, Y.-P. (1994) Botanical diversity and inventory of Taiwan. *In*: **Peng, C.-I** & Chou, C.-H. (eds.) *Biodiversity and Terrestrial Ecosystem*. Institute of Botany, Academia Sinica, Taipei, pp. 78–85.

49. Hsiao, S.-C., Mauseth, J.-D. & **Peng, C.-I** (1995) Composite bundles, the host/parasite interface in the holoparasitic angiosperms *Langsdorffia* and *Balanophora* (Balanophoraceae). *American Journal of Botany* 82: 81–91.

50. Hetterscheid, W. L. A. & **Peng, C.-I** (1995) Notes on the genus *Amorphophallus* (Araceae) IV. Revision of the species in Taiwan. *Botanical Bulletin of Academia Sinica* 36: 101–112.

51. **Peng, C.-I**, Tobe, H. & Takahashi, M. (1995) Reproductive morphology and relationships of *Triplostegia* (Dipsacales). *Botanische Jahrbücher für Systematik* 116: 505–516.

52. Yang, K.-C. & **Peng, C.-I** (1996) *Arabis serrata* Franch. et Sav. (Brassicaceae), a newly recorded alpine plant in Taiwan. *Taiwan Journal of Forest Science* 11: 229–231.

53. 彭鏡毅 (ed.) (1996) *台灣維管束植物編碼索引（初版）*. 行政院農業委員會，台北市, 254 pp. [**Peng, C.-I** (ed.) (1996) *Index to Codes of Vascular Plants of Taiwan*. Council of Agriculture, Taipei, 254 pp.]

54. 蔣志剛 & 彭鏡毅. (1997) 第23章：台灣的生物多樣性及其保護. *In*: 蔣志剛，馬克平 & 韓興國 (eds.) *保護生物學*. 浙江科學技術出版社，杭州市, pp. 229–234. [Jiang, Z.-G. & **Peng, C.-I** (1997) Chapter 23. Biodiversity and Conservation in Taiwan. In: Jiang, Z.-G. & Ma, K.-P. (eds.) *Conservation Biology*. Zhejiang Scientific and Technology Press, pp. 229–234.]

55. Kita, Y., Ito, M. & **Peng, C.-I** (1997) Phylogenetic position of Taiwanese and Korean aconites, *Aconitum bartletii*, *A. napiforme* and *A. jaluense* (Ranunculaceae). *Journal of Phytogeography and Taxonomy* 45: 75–82.

56. Tokuoka, T. & **Peng, C.-I** (1997) Flora morphology and its systematic implications in *Drypetes integerrima* (Koidz.) Hosok. (Euphorbibaceae, tribe Drypeteae) from Bonin Islands, Japan. *Acta Phytotaxonomica et Geobotanica* 48: 159–166.

57. **Peng, C.-I** & Lowry, P. P. I. (eds.) (1998) *Rare, Threaten, and Endangered Flora of Asia and Pacific Rim*. Institute of Botany, Academia Sinica, Taipei, 283 pp.

58. **Peng, C.-I** & Lammers, T. G. (1998) *Triodanis* Raf. (Campanulaceae : Campanuloideae), a new generic record for the flora of Taiwan. *Botanical Bulletin of Academia Sinica* 39: 213–216.

59. 邱少婷 & 彭鏡毅 (eds.) (1998) *海峽兩岸植物多樣性與保育*. 國立自然科學博物館，台中市, 327 pp. [Chiu, S.-T. & **Peng, C.-I** (eds.) (1998) *Proceedings of the Cross-Strait Symposium on Floristic Diversity and Conservation*. National Museum of Natural Sciences, Taichung, 327 pp.]

60. 蔣鎮宇，彭鏡毅, Schaal, B. A. & 許再文. (1998) 台灣特有天南星科植物密毛魔芋遺傳變異及保育之探討. *自然保育季刊* 23: 42–49. [Chiang, T.-Y., **Peng, C.-I**, Schaal, B. A. & Hsu, T.-W. (1998) Genetic variation and conservation in *Amorphophallus* hirta (Araceae), a plant endemic to Taiwan. *Nature Conservation Quarterly* 23: 42–49.]

61. Chiang, T.-Y., Schaal, B. A. & **Peng, C.-I** (1998) Universal primers for amplification and sequencing a noncoding spacer between the *atp*B and *rbc*L genes of chloroplast DNA. *Botanical Bulletin of Academia Sinica* 39: 245–250.

62. **Peng, C.-I**, Chen, C.-H., Leu, W.-P. & Yen, H.-F. (1998) *Pluchea* Cass. (Asteraceae:

Inuleae) in Taiwan. *Botanical Bulletin of Academia Sinica* 39: 287–297. (A-6)
63. Soejima, A. & **Peng, C.-I** (1998) Cytological features of the *Aster ageratoides* complex (Asteraceae) in Taiwan. *Botanical Bulletin of Academia Sinica* 39: 299–302. (A-7)
64. **Peng, C.-I** & Yang, K.-C. (1998) Unwelcome naturalization of *Chromolaena odorata* (Asteraceae) in Taiwan. *Taiwania* 43: 289–294. (A-8)
65. **Peng, C.-I**, Chung, K.-F. & Leu, W.-P. (1998) Notes on three newly naturalized plants (Asteraceae) in Taiwan. *Taiwania* 43: 320–329. (A-9)
66. Liu, S.-L., Chiang, T.-Y. & **Peng, C.-I** (1998) Sequence announcement (Promoter of *rbcL* gene from *Begonia nantoensis*). *Plant Molecular Biology* 38: 905. (*B-4*)
67. Liu, S.-L., Chiang, T.-Y. & **Peng, C.-I** (1998) Sequence announcement (Promoter of *rbcL* gene from *Begonia chitoensis*). *Plant Molecular Biology* 38: 907. (*B-5*)
68. Editorial Committee of the Flora of Taiwan, Second Edition (ed.) (1998) *Flora of Taiwan, 2nd edn. Vol 4*. Department of Botany, National Taiwan University, Taipei, 1217 pp. [Executive Editor]
69. Kao, M.-T. & **Peng, C.-I** (1998) Primulaceae. *In*: Editorial Committee of the Flora of Taiwan Second Edition (ed.) *Flora of Taiwan, 2nd edn. Vol. 4*. Editorial Committee of the Flora of Taiwan, 2nd edn., Taipei, pp. 57–72.
70. Li, H.-L. & **Peng, C.-I** (1998) Loganiaceae. *In*: Editorial Committee of the Flora of Taiwan Second Edition (ed.) *Flora of Taiwan, 2nd edn. Vol. 4*. Editorial Committee of the Flora of Taiwan, 2nd edn., Taipei, pp. 89–98.
71. D'Arcy, W. G. & **Peng, C.-I** (1998) Solanaceae. *In*: Editorial Committee of the Flora of Taiwan Second Edition (ed.) *Flora of Taiwan, 2nd edn. Vol. 4*. Editorial Committee of the Flora of Taiwan, 2nd edn., Taipei, pp. 549–582.
72. **Peng, C.-I**, Chung, K.-F. & Li, H.-L. (1998) Compositae. *In*: Editorial Committee of the Flora of Taiwan Second Edition (ed.) *Flora of Taiwan, 2nd edn. Vol. 4*. Editorial Committee of the Flora of Taiwan, 2nd edn., Taipei, pp. 807–1101. (A-10)
73. **Peng, C.-I** & Hu, C.-M. (1999) *Lysimachia chingshuiensis* (Primulaceae), a new species from eastern Taiwan. *Botanical Bulletin of Academia Sinica* 40: 49–52.
74. **Peng, C.-I** & Leu, W.-P. (1999) Novelties in Asteraceae of Taiwan: *Blumea linearis* and *Senecio tarokoensis*. *Botanical Bulletin of Academia Sinica* 40: 53–60. (A-11)
75. **Peng, C.-I**, Liu, S.-L. & Chiang, T.-Y. (1999) Conservation of *Ludwigia* × *taiwanensis* (Onagraceae) in Taiwan. *Endemic Species Research* 10: 73–78. (O-14)
76. 蔣鎮宇 & 彭鏡毅. (1999) 台灣特有植物的親緣地理 *特有生物保育研討會論文集*. 特有生物研究保育中心出版，南投縣, pp. 148–155. [Chiang, T.-Y. & **Peng, C.-I** (1999) Phylogeography of the endemic plants of Taiwan *Proceedings of the Symposium on Conservation of Endemic Organisms*. Taiwan Endemic Species Research Center, Chichi, pp. 148–155.]
77. Kondo, K., **Peng, C.-I**, Aoyama, M. & Tanaka, R. (1999) Chromosome studies in chrysanthemum flora of Taiwan 1. *Dendranthema horaimontana* (Masam.) S.S.Ying and *D. morii* (Hayata) Kitam. *Chromosome Science* 3: 49–54. (A-12)

78. Naruhashi, N., Iwatsubo, Y. & **Peng, C.-I** (1999) Cytology, flower morphology and distribution of *Fragaria hayatai* Makino (Rosaceae). *Journal of Phytogeography and Taxonomy* 47: 139–143.

79. 楊遠波，劉和義，彭鏡毅，施炳霖 & 呂勝由 (eds.) (1999) *台灣維管束植物簡誌，第四卷*. 行政院農業委院會，台北市, 422 pp. [Yang, Y.-P., Liu, H.-Y., **Peng, C.-I**, Shih, B.-L. & Lu, S.-Y. (eds.) (2000) *Manual of Taiwan Vascular Plants, Vol. 4.* Council of Agriculture, Taipei, 422 pp.]

80. 彭鏡毅 & 鍾國芳. (1999) 菊科. *In*: 楊遠波，劉和義，彭鏡毅，施炳霖 & 呂勝由 (eds.) *台灣維管束植物簡誌，第四卷*. 行政院農業委院會，台北市, pp. 215–290. [**Peng, C.-I** & Chung, K.-F. (2000) Compositae (Asteraceae). In: Yang, Y.-P., Liu, H.-Y., **Peng, C.-I**, Shih, B.-L. & Lu, S.-Y. (eds.) *Manual of Taiwan Vascular Plants, Vol. 4.* Council of Agriculture, Taipei, pp. 215–290.] (A-13)

81. Chou, C.-H., Chen, T.-Y., Liao, C.-C. & **Peng, C.-I** (2000) Long-term ecological research in the Yuanyang Lake forest ecosystem I. Vegetation composition and analysis. *Botanical Bulletin of Academia Sinica* 41: 61–72.

82. **Peng, C.-I** & Sue, C.-Y. (2000) *Begonia ×taipeiensis* (Begoniaceae), a new natural hybrid in Taiwan. *Botanical Bulletin of Academia Sinica* 41: 151–158. (*B-6*)

83. **Peng, C.-I** & Chiang, T.-Y. (2000) Molecular confirmation of unidirectional hybridization in *Begonia ×taipeiensis* Peng (Begoniaceae) from Taiwan. *Annals of the Missouri Botanical Garden* 87: 273–285. (*B-7*)

84. Al-Shehbaz, I. A. & **Peng, C.-I** (2000) The genus *Barbarea* (Brassicaceae) in Taiwan. *Botanical Bulletin of Academia Sinica* 41: 237–242.

85. Yang, S.-Z. & **Peng, C.-I** (2000) Two re-discovered plants in Taiwan: *Hydrolea zeylanica* (L.) Vahl and *Cyanotis axillaris* (L.) D. Don ex Sweet. *Bulletin of National Pingtung University of Science and Technology* 9: 199–204.

86. 洪國祥，呂勝由，彭鏡毅 & 蔣鎮宇. (2000) 巒大杉及福州杉的親源地理及保育. *自然保育季刊* 31: 33–36. [Hong, K.-H., Lu, S.-Y., **Peng, C.-I** & Chiang, T.-Y. (2000) Phylogeography and conservation of *Cunninghamia konishii* and C. *lanceolata* (Cupressaceae). *Nature Conservation Quarterly* 31: 33–36.]

87. 彭鏡毅. (2000) 菊科. In: 呂勝由，邱文良 & 鄭育斌 (eds.) *臺灣稀有及瀕危植物之分級彩色圖鑑 (V)*. 行政院農業委員會，臺北市, pp. 41–42, 51–58, 61–64, 67–70, 75–80, 85–88, 91–92, 95–96, 99–100, 107–108, 111–116, 119–120, 131–132, 139–142. [Peng, C.-I (2000) Asteraceae. *In*: Lu, S.-Y., Chiou, W.-L. & Cheng, Y.-P. (eds.) *Rare and Endangered Plants in Taiwan (V)*. Council of Agriculture, Executive Yuan, Taipei, pp. pp. 41–42, 51–58, 61–64, 67–70, 75–80, 85–88, 91–92, 95–96, 99–100, 107–108, 111–116, 119–120, 131–132, 139–142.] (A-14)

88. 彭鏡毅 & 胡維新. (2000) 美國密蘇里植物園的科學研究和環境教育. *In*: 嚴新富 (ed.) *植物園資源及經營管理：植物園資源及經營管理學術研討會論文集*. 國立自然科學博物館，台中市, pp. 197–211. [**Peng, C.-I** & Hu, W.-H. (2000) Scientific researches and environmental education of the Missouri Botanical Garden*, USA. *In*: Yen, H.-F. (ed.)

Proceedings of the Symposium of Resources of Botanical Garden and Management. National Museum of Natural Science, Taichung, pp. 197–211.]

89. **Peng, C.-I**, Chen, Y.-J. & Wang, J.-C. (2000) Notes on Commelinaceae of Taiwan: *Cyanotis* and *Belosynapsis,* and rediscovery of *Murdannia edulis*. *Biological Bulletin of National Taiwan Normal University* 35: 77–93.

90. Wang, J.-C., Chen, J.-J. & **Peng, C.-I** (2000) Commelinaceae. *In*: Editorial Committee of the Flora of Taiwan, n.e. (ed.) *Flora of Taiwan, 2nd edn. Vol. 5*. Editorial Committee of the Flora of Taiwan, 2nd edn., Taipei, pp. 153–178.

91. **Peng, C.-I** (2000) *Amorphophallus* Blume *ex* Decne. *In*: Editorial Committee of the Flora of Taiwan, n.e. (ed.) *Flora of Taiwan, 2nd edn. Vol. 5*. Editorial Committee of the Flora of Taiwan, 2nd edn., Taipei, pp. 669–672.

92. D'Arcy, W. G., Keating, R. C., Zhang, Z.-Y. & **Peng, C.-I** (2001) The genus *Tubocapsicum* (Solanaceae). *Botanical Bulletin of Academia Sinica* 42: 67–84.

93. Yang, S.-Z. & **Peng, C.-I** (2001) An invading plant in Taiwan—*Mimosa pigra* L. *Quarterly Journal of Forest Research* 23: 1–6.

94. Chiang, T.-Y., Hong, K.-H. & **Peng, C.-I** (2001) Experimental hybridization reveals biased inheritance of the internal transcribed spacer of the nuclear ribosomal DNA in *Begonia* ×*taipeiensis*. *Journal of Plant Research* 114: 343–351. (*B-8*)

95. Lu, S.-Y., **Peng, C.-I**, Cheng, Y.-P., Hong, K.-H. & Chiang, T.-Y. (2001) Chloroplast DNA phylogeography of *Cunninghamia konishii* (Cupressaceae), an endemic conifer of Taiwan. *Genome* 44: 797–807.

96. **Peng, C.-I** & Lin, Y.-L. (2001) Evaluation of medicinal plants of Asteraceae in Taiwan *Proceedings of International Symposium of Plant Biodiversity and Development of Bioactive Natural Products*. National Museum of Natural Science, Taichung, pp. 117–125. (A-15)

97. Suzuki, R., Kondo, K. & **Peng, C.-I** (2001) Chromosome studies in chrysanthemum flora of Taiwan 2. *Ajania pacifica* (Nakai) Bremer et Humpnhries dwarf form, *Crossostephium chinense* (L.) Makino, *Dendranthema arisanense* (Hayata) Ling et Shih, *D. indica* (L.) Des Moul. and *D. lavandulifolia* (Fisch ex Trautv.) Ling et Shih var. *tomentellum* (Hand.-Mazz.) Ling et Shih. *Chromosome Science* 5: 57–62. (A-16)

98. Hsing, Y.-I. C., Hsieh, J.-S., **Peng, C.-I**, Chou, C.-H. & Chiang, T.-Y. (2001) Systematic status of the *Glycine tomentella* and *G. tabacina* species complexes (Fabaceae) based on ITS sequences of nuclear ribosomal DNA. *Journal of Plant Research* 114: 435–442.

99. Anderberg, A. A., **Peng, C.-I**, Trift, I. & Källersjö, M. (2001) The *Stimpsonia* problem: evidence from DNA sequences of plastid gene *atpB*, *ndhF* and *rbcL*. *Botanische Jahrbücher für Systematik* 123: 369–376.

100. Chung, S.-W. & **Peng, C.-I** (2002) *Senecio kuanshanensis* (Asteraceae), a new species from southern Taiwan. *Botanical Bulletin of Academia Sinica* 43: 155–159. (A-17)

101. Oginuma, K. & **Peng, C.-I** (2002) Karyomorphology of Taiwanese *Begonia* (Begoniaceae): taxonomic implications. *Journal of Plant Research* 115: 225–235. (*B-9*)

102. Naruhashi, N., Iwatsubo, Y. & **Peng, C.-I** (2002) Chromosome numbers in *Rubus* (Rosaceae) of Taiwan. *Botanical Bulletin of Academia Sinica* 43: 193–201.

103. Shui, Y.-M., **Peng, C.-I** & Wu, C.-Y. (2002) Synopsis of the Chinese species of *Begonia* (Begoniaceae), with a reappraisal of sectional delimitation. *Botanical Bulletin of Academia Sinica* 43: 313–327. (*B-10*)

104. 彭鏡毅 & 穆家宏. (2002) 國內植物資料庫之整合與發展. *In*: 周明 & 黃寬重 (eds.) *博物館典藏數位再造理論與實務研討會－人與自然論文集*. 數位典藏國家型科技計畫內容發展分項計畫出版，台北市, pp. 85–90. [**Peng, C.-I** & Mu, C.-H. (2002) Integration and development of domestic plant databases. *In*: Chou, M. & Huang, K.-C. (eds), *Symposium on theories and practices of digital archive for reconstructing the museums—humanity and nature*. National Museum of Natural Science, Taichung, pp. 85–90.]

105. 彭鏡毅譯 (Peter H. Raven 撰). (2002) 生物多樣性與永續發展. *自然保育季刊* 40: 6–15. [Raven, P. H. (2002) Biodiversity and sustainable development. *Nature Conservation Quarterly* 40: 6–15. Translated into Chinese by **Peng, C.-I**]

106. 許再文 & 彭鏡毅. (2002) 台灣的茄科植物及其相關研究介紹. *自然保育季刊* 40: 28–35. [Hsu, T.-W. & **Peng, C.-I** (2002) Solanaceae of Taiwan and its related researches. *Nature Conservation Quarterly* 40: 28–35.]

107. Editorial Committee of the Flora of Taiwan, Second Edition (ed.) (2003) *Flora of Taiwan, 2nd edn. Vol. 6*. Department of Botany, National Taiwan University, Taipei, 343 pp. [Editor]

108. 彭鏡毅 (ed.) (2003) *台灣維管束植物編碼索引（2003年6月增編修訂）*. 行政院農業委員會，台北市, 276 pp. [**Peng, C.-I** (2003) *Revised Index to Codes of Vascular Plants of Taiwan*, Council of Agriculture, Taipei, 276 pp.]

109. Yukawa, T., Chung, S.-W., Luo, Y.-B., **Peng, C.-I**, Momohara, A. & Setoguchi, H. (2003) Reappraisal of *Kitigorchis* (Orchidaceae). *Botanical Bulletin of Academia Sinica* 44: 345–351.

110. 彭鏡毅 & 呂文賓. (2003) *鴛鴦湖自然保留區常見植物解說手冊 (一)*. 行政院退輔會榮民森林保育處、中央研究院植物研究所，台北市, 155 pp. [**Peng, C.-I** and W. P. Leu. 2003. Guide to Plants of Yuanyang Lake Nature Reserve. Council of Agriculture, Taiwan and Academia Sinica, Taiwan, 155 pp.]

111. Tseng, Y.-H. & **Peng, C.-I** (2004) *Ambrosia psilostachya* DC. (Asteraceae) a newly naturalized plant in Taiwan. *Endemic Species Research* 6: 71–74. (A-18)

112. Kokubugata, G., **Peng, C.-I** & Yokota, M. (2004) Comparison of karyotypes among three *Heloniopsis* species (Liliaceae) from Ryukyu Archipelago and Taiwan. *Annals of the Tsukuba Botanical Garden* 23: 13–16.

113. 彭鏡毅. (2004) *發現臺灣植物套書*. 中央研究院植物研究所/生物多樣性研究中心，台北市(http://taiwanplants.ndap.org.tw/) [**Peng, C.-I** (2004) *Discover Plants of Taiwan*. Institute of Botany and Biodiversity Research Center, Academia Sinica, Taipei.]

114. Ye, H.-G., Wang, F.-G., Ye, Y.-S. & **Peng, C.-I** (2004) *Begonia coptidifolia* (Begoniaceae), a new species from China. *Botanical Bulletin of Academia Sinica* 45: 259–266. (*B-11*)

115. Hsu, T.-W., Ku, S.-M. & **Peng, C.-I** (2004) *Persicaria capitata* (Buchanan-Hamilton *ex* D.

Don) H. Gross (Polygonaceae), a newly naturalized plant in Taiwan. *Taiwania* 49: 183–187.

116. Tsai, C.-C., **Peng, C.-I**, Huang, S.-C., Huang, P.-L. & Chou, C.-H. (2004) Determination of the genetic relationship of *Dendrobium* species (Orchidaceae) in Taiwan based on the sequence of the internal transcribed spacer of ribosomal DNA. *Scientia Horticulturae* 101: 315–325.

117. Ku, S.-M., **Peng, C.-I** & Liu, Y. (2004) Notes on *Begonia* (sect. *Coelocentrum*, Begoniaceae) from Guangxi, China, with the report of two new species. *Botanical Bulletin of Academia Sinica* 45: 353–367. (*B-12*)

118. Kokubugata, G. & **Peng, C.-I** (2004) Comparison of floral morphologies in *Conandron ramondioides* (Gesneriaceae) from Japan and Taiwan. *Edinburgh Journal of Botany* 61: 21–30.

119. **Peng, C.-I**, Shui, Y.-M., Liu, Y. & Ku, S.-M. (2005) *Begonia fangii* (sect. *Coelocentrum*, Begoniaceae), a new species from limestone areas in Guangxi, China. *Botanical Bulletin of Academia Sinica* 46: 83–89. (*B-13*)

120. Hsu, T.-W., Chiang, T.-Y. & **Peng, C.-I** (2005) *Lepidium bonariense* L. (Brassicaceae) newly naturalized to Taiwan. *Endemic Species Research* 7: 89–94.

121. Huang, S.-C., Wang, W.-K., **Peng, C.-I** & Chiang, T.-Y. (2005) Phylogeography and conservation genetics of *Hygrophila pogonocalyx* (Acanthaceae) based on *atpB-rbcL* noncoding spacer cpDNA. *Journal of Plant Research* 118: 1–11.

122. **Peng, C.-I**, Ku, S.-M. & Leong, W.-C. (2005) *Begonia liuyanii* (sect. *Coelocentrum*, Begoniaceae), a new species from limestone areas in Guangxi, China. *Botanical Bulletin of Academia Sinica* 46: 245–254. (*B-14*)

123. **Peng, C.-I**, Chen, Y.-K. & Leong, W.-C. (2005) Five new species of *Begonia* (Begoniaceae) from Taiwan. *Botanical Bulletin of Academia Sinica* 46: 255–272. (*B-15*)

124. **Peng, C.-I**, Schmidt, C. L., Hoch, P. C. & Raven, P. H. (2005) Systematics and evolution of *Ludwigia* section *Dantia* (Onagraceae). *Annals of the Missouri Botanical Garden* 92: 307–359. (O-15)

125. Liu, Y., Ku, S.-M. & **Peng, C.-I** (2005) *Begonia picturata* (sect. *Coelocentrum*, Begoniaceae), a new species from limestone areas in Guangxi, China. *Botanical Bulletin of Academia Sinica* 46: 367–376. (*B-16*)

126. Li, H.-Z., Ma, H., Guan, K.-Y. & **Peng, C.-I** (2005) *Begonia rubinea* (sect. *Platycentrum*, Begoniaceae), a new species from Guizhou, China. *Botanical Bulletin of Academia Sinica* 46: 377–383. (*B-17*)

127. Chung, K.-F., **Peng, C.-I**, Downie, S. R., Spalik, K. & Schaal, B. A. (2005) Molecular systematics of the trans-Pacific alpine genus *Oreomyrrhis* (Apiaceae): Phylogenetic affinities and biogeographic implications. *American Journal of Botany* 92: 2054–2071.

128. 陳怡雁, 彭鏡毅, 許再文 & 蔣鎮宇. (2005) 探索島嶼瀕危植物－鈴木草屬的天空. *自然保育季刊* 52: 30–35. [Chen, Y.-Y., **Peng, C.-I**, Hsu, T.-W. & Chiang, T.-Y. 2005. Endangered insular plant: the genus Suzukia. *Nature Conservation Quarterly* 52: 30–35.]

129. Hsu, T.-W., Chiang, T.-Y. & **Peng, C.-I** (2006) *Croton bonplandianus* Baillon

(Euphorbiaceae), a plant newly naturalized to Taiwan. *Endemic Species Research* 8: 77–82.

130. **Peng, C.-I**, Leong, W.-C. & Shui, Y.-M. (2006) Novelties in *Begonia* sect. *Platycentrum* for China: *B. crocea*, sp nov and *B. xanthina* Hook., a new distributional record. *Botanical Studies* 47: 89–96. (*B-18*)
131. Fang, D., Ku, S.-M., Wei, Y.-G., Qin, D.-H. & **Peng, C.-I** (2006) Three new taxa of *Begonia* (sect. *Coelocentrum*, Begoniaceae) from limestone areas in Guangxi, China. *Botanical Studies* 47: 97–110. (*B-19*)
132. Hsu, T.-W., **Peng, C.-I** & Wang, C.-M. (2006) *Austroeupatorium inulifolium* (Kunth) King & Robinson, a newly naturalized plant in Taiwan. *Taiwania* 51: 41–45. (A-19)
133. Ku, S.-M., Liu, Y. & **Peng, C.-I** (2006) Four new species of *Begonia* sect. *Coelocentrum* (Begoniaceae) from limestone areas in Guangxi, China. *Botanical Studies* 47: 207–222. (*B-20*)
134. **Peng, C.-I**, Leong, W.-C., Ku, S.-M. & Liu, Y. (2006) *Begonia pulvinifera* (sect. *Diploclinium*, Begoniaceae), a new species from limestone areas in Guangxi, China. *Botanical Studies* 47: 319–327. (*B-21*)
135. Setoguchi, H., Yukawa, T., Tokuoka, T., Momohara, A., Sogo, A., Takaso, T. & **Peng, C.-I** (2006) Phylogeography of the genus *Cardiandra* based on genetic variation in cpDNA sequences. *Journal of Plant Research* 119: 401–405.
136. Setoguchi, H., Yukawa, T., Tokuoka, T., Momohara, A., Sogo, A., Takaso, T. & **Peng, C.-I** (2006) Comparison of leaf and flora morphology among insular endemic of *Pieris* (Eriaceae) on the Ryukyu Islands and Taiwan. *Acta Phytotaxonomica et Geobotanica* 57: 173–182.
137. Kokubugata, G., **Peng, C.-I** & Madulid, D. A. (2006) Chromosome number of *Lobelia nummularia* (Campanulaceae) in Taiwan and the Philippines. *Annals of the Tsukuba Botanical Garden* 25: 7–10.
138. Yang, T. Y. A., Chiang, T.-Y., **Peng, C.-I** & Hsu, T.-W. (2006) *Choranthus henryi* Hemsl. (Chloranthaceae), a new record to the flora of Taiwan. *Taiwania* 51: 283–286.
139. Kokubugata, G., **Peng, C.-I**, Saito, Y., Yokota, M. & Kobayashi, S. (2006) Taxonomic reconsideration of *Lysimachia ardisioides* (Primulaceae) from Taiwan. *Memoirs of the National Science Museum* 44: 135–140.
140. **Peng, C.-I**, Hsieh, T.-Y. & Ngyuen, Q. H. (2007) *Begonia kui* (sect. *Coelocentrum*, Begoniaceae), a new species from Vietnam. *Botanical Studies* 48: 127–132. (*B-22*)
141. **Peng, C.-I**, Boufford, D. E., Takaso, T. & Chiang, T.-Y. (2007) *A Selection of Plants from Iriomote Island, Japan*. Endemic Species Research Center & Research Center for Biodiversity, Nantou & Taiwan, 183 pp.
142. Hsu, T.-W., Chiang, T.-Y. & **Peng, C.-I** (2007) *Rubus amphidasys* Focke (Rosaceae): a newly recorded plant in Taiwan. *Taiwania* 52: 113–116.
143. Liu, Y., **Peng, C.-I** & Yang, Q.-E. (2007) Validation of the name *Parasenecio morrisonensis* (Compositae-Senecioneae) for a species endemic to Taiwan. *Taxon* 56: 583–584. (A-20)

144. Gu, C.-Z., **Peng, C.-I** & Turland, N. J. (2007) Begoniaceae. *In*: Wu, Z.-Y., Raven, P.H. & Hong, D.-Y. (eds.) *Flora of China, vol. 13*. Science Press and Missouri Botanical Garden, Beijing and St. Louis, pp. 153–207. (*B-23*)

145. Hsu, T.-W., **Peng, C.-I**, Peng, J.-J. & Chiang, T.-Y. (2007) Confirmation of the distribution of *Solanum miyakojimaense* Yamazaki & Takushi (Solanaceae) in Taiwan. *Taiwania* 52: 190–193.

146. Hsieh, T.-Y., Hsu, T.-C., Kono, Y., Ku, S.-M. & **Peng, C.-I** (2007) *Gentiana bambuseti* (Gentianaceae), a new species from Taiwan. *Botanical Studies* 48: 349–355.

147. **Peng, C.-I**, Tiang, C.-L. & Hsu, T.-W. (2007) *Tricyrtis ravenii* (Liliaceae), a new species from Taiwan. *Botanical Studies* 48: 357–364.

148. Yan, H.-F., **Peng, C.-I**, Hu, C.-M. & Hao, G. (2007) Phylogeographic structure of *Primula obconica* (Primulaceae) inferred from chloroplast microsatellites (cpSSRs) markers. *Acta Phytotaxonomica Sinica* 45: 488–496.

149. Liu, Y., Ku, S.-M. & **Peng, C.-I** (2007) *Begonia bamaensis* (sect. *Coelocentrum*, Begoniaceae), a new species from limestone areas in Guangxi, China. *Botanical Studies* 48: 465–473. (*B-24*)

150. Oginuma, K., Sato, H., Kono, Y., Chen, S., Zhou, Z., **Peng, C.-I**, Momohara, A., Yukawa, T. & Setoguchi, H. (2007) Intraspecific polyploidy of *Houttuynia cordata* and evolution of chromosome number in the Saururaceae. *Chromosome Botany* 2: 87–91.

151. Chung, K.-F., Kono, Y., Wang, C.-M. & **Peng, C.-I** (2008) Notes on *Acmella* (Asteraceae: Heliantheae) in Taiwan. *Botanical Studies* 49: 73–82. (A-21)

152. **Peng, C.-I**, Liu, Y. & Ku, S.-M. (2008) *Begonia aurantiflora* (sect. *Coelocentrum*, Begoniaceae), a new species from limestone areas in Guangxi, China. *Botanical Studies* 49: 83–92. (*B-25*)

153. Mitsui, Y., Chen, S.-T., Zhou, Z.-K., **Peng, C.-I**, Deng, Y.-F. & Setoguchi, H. (2008) Phylogeny and biogeography of the genus *Ainsliaea* (Asteraceae) in the Sino-Japanese region based on nuclear rDNA and plastid DNA sequence data. *Annals of Botany* 101: 111–124. (A-22)

154. Setoguchi, H., Watanabe, W., Maeda, Y. & **Peng, C.-I** (2008) Molecular phylogeny of the genus *Pieris* (Ericaceae) with special reference to phylogenetic relationships of insular plants on the Ryukyu Islands. *Plant Systematics and Evolution* 270: 217–230.

155. Tseng, Y.-H., Wang, C.-M. & **Peng, C.-I** (2008) *Clibadium surinamense* L. (Asteraceae): a newly naturalized plant in Taiwan. *Taiwania* 53: 103–106. (A-23)

156. Kokubugata, G., Madulid, D. A. & **Peng, C.-I** (2008) Notes on *Lysimachia* sect. *Idiophyton* (Primulaceae) in the Philippines and Taiwan. *Memoirs of the National Museum of Nature and Science* 45: 121–125.

157. 朱恩良, 楊智凱 & 彭鏡毅. (2008) 臺灣產金縷梅科植物. *自然保育季刊* 63: 31–37. [Zhu, E.-L., Yang, C.-K. & **Peng, C.-I** (2008) Hamamelidaceae of Taiwan. *Nature Conservation Quarterly* 63: 31–37.]

158. 鍾明哲, 鐘詩文 & 彭鏡毅. (2008) 台灣菊科新成員彙整. *自然保育季刊* 63: 45–51.

[Chung, M.-J., Chung, S.-W. & **Peng, C.-I** (2008) Compilation of new members of Asteraceae in Taiwan. *Nature Conservation Quarterly* 63: 45–51.] (A-24)

159. Tseng, Y.-H., Liou, C.-Y. & **Peng, C.-I** (2008) *Helianthus debilis* Nuttall subsp. *cucumerifolius* (Torrey & A. Gray) Heiser (Asteraceae): a newly naturalized plant in Taiwan. *Taiwania* 53: 316–320. (A-25)

160. **Peng, C.-I**, Ku, S.-M., Kono, Y., Chung, K.-F. & Liu, Y. (2008) Two new species of *Begonia* (sect. *Coelocentrum*, Begoniaceae) from limestone areas in Guangxi, China: *B. arachnoidea* and *B. subcoriacea*. *Botanical Studies* 49: 405–418. (*B-26*)

161. 彭鏡毅, 楊智凱 & 古訓銘. (2008) 演化路上的台北秋海棠. *科學人雜誌* 82: 116–117. [**Peng, C.-I**, C. K. Yang and S. M. Ku. 2008. *Begonia* × *taipeiensis* on the road of evolution. Sci. Amer. (Chin. ver.) 82: 116–117.] (*B-27*)

162. 邵廣昭, 彭鏡毅 & 吳文哲 (eds.) (2008) *2008臺灣物種多樣性—I. 研究現況.* 農委會林務局, 臺北市, 373 pp.

163. 邵廣昭, 彭鏡毅 & 吳文哲 (eds.) (2008) *2008臺灣物種多樣性—II. 物種名錄.* 農委會林務局, 臺北市, 796 pp.

164. Tseng, Y.-H., Liou, C.-Y., Yen, H.-F. & **Peng, C.-I** (2008) *Flaveria bidens* (L.) Kuntze (Asteraceae), a newly naturalized plant in Taiwan. *Quarterly Journal of Forest Research* 63: 45–51. (A-26)

165. Iwashina, T., Saito, Y., **Peng, C.-I**, Yokota, M. & Kokubugata, G. (2008) Foliar flavonoids from two *Begonia* species in Japan. *Bulletin of the National Museum of Nature and Science. Series B, Botany* 34: 175–181. (*B-28*)

166. Shen, M.-Y., **Peng, C.-I** & Hsu, T.-W. (2009) *Veronica hederifolia* L. (Scrophulariaceae) newly naturalized to Taiwan. *Endemic Species Research* 11: 47–50.

167. 楊智凱, 胡嘉穎, 游旨价 & 彭鏡毅. (2009) 錐麓古道的明珠－大斷崖山地區之稀有植物資源. *自然保育季刊* 65: 45–51 [Yang, C.-K., Hu, C.-Y, Yu, C.-C. & **Peng, C.-I** (2009) Bright pearls on Zhuilu Ancient Trail: rare plant resources in Daduanyashan area. *Nature Conservation Quarterly* 65: 45–51.]

168. Jung, M.-J., Hsu, T.-C., Chung, S.-W. & **Peng, C.-I** (2009) Three newly naturalized Asteraceae plants in Taiwan. *Taiwania* 54: 76–81. (A-27)

169. **Peng, C.-I** & Ku, S.-M. (2009) *Begonia ×chungii* (Begoniaceae), a new natural hybrid in Taiwan. *Botanical Studies* 50: 241–250. (*B-29*)

170. Hou, M.-F., Liu, Y., Kono, Y. & **Peng, C.-I** (2009) *Aspidistra daxinensis* (Ruscaceae), a new species from limestone areas in Guangxi, China. *Botanical Studies* 50: 371–378.

171. Saito, Y., Iwashina, T., **Peng, C.-I** & Kokubugata, G. (2009) Taxonomic reconsideration of *Disporum luzoniense* (Liliaceae s.l.) using flavonoid characters. *Blumea* 54: 59–62.

172. Hung, K.-H., Schaal, B. A., Hsu, T.-W., Chiang, Y.-C., **Peng, C.-I** & Chiang, T.-Y. (2009) Phylogenetic relationships of diploid and polyploid species in *Ludwigia* sect. *Isnardia* (Onagraceae) based on chloroplast and nuclear DNAs. *Taxon* 58: 1216–1225. (O-16)

173. 朱恩良, 楊智凱 & 彭鏡毅. (2009) 山寒水瘦唯櫻草，趕在梅花先報春—臺灣的報春花科植物. *自然保育季刊* 68: 3–11. [Zhu, E.-L., Yang, C.-K. & **Peng, C.-I** (2009)

Primulaceae of Taiwan. *Nature Conservation Quarterly* 68: 3–11.]

174. Chung, K.-F., Ku, S.-M., Kono, Y. & **Peng, C.-I** (2009) *Emilia praetermissa* Milne-Redh. (Asteraceae)—A misidentified alien species in northern Taiwan. *Taiwania* 54: 385–390. (A-28)

175. Nguyen, Q. H., **Peng, C.-I** & Ku, S.-M. (2010) *Begonia vietnamensis*, an attractive new species with peltate leaves from Vietnam. *The Begonian* 77: 18–20. (*B-30*)

176. **Peng, C.-I**, Liu, Y., Ku, S.-M., Kono, Y. & Chung, K.-F. (2010) *Begonia* ×*breviscapa* (Begoniaceae), a new intersectional natural hybrid from limestone areas in Guangxi, China. *Botanical Studies* 51: 107–117. (*B-31*)

177. Kokubugata, G., Nakamura, K., Shinohara, W., Saito, Y., **Peng, C.-I** & Yokota, M. (2010) Evidence of three parallel evolutions of leaf dwarfism and phytogeography in *Lysimachia* sect. *Nummularia* in Japan and Taiwan. *Molecular Phylogenetics and Evolution* 54: 657–663.

178. Iwashina, T., **Peng, C.-I** & Kokubugata, G. (2010) Flavone *O*- and *C-glycosides* from *Pothos chinensis* (Araceae). *Bulletin of the National Museum of Nature and Science. Series B, Botany* 36: 27–32.

179. Yan, H.-F., He, C.-H., **Peng, C.-I**, Hu, C.-M. & Hao, G. (2010) Circumscription of *Primula* subgenus *Auganthus* (Primulaceae) based on chloroplast DNA sequences. *Journal of Systematics and Evolution* 48: 123–132.

180. Chung, K.-F., van der Werff, H. & **Peng, C.-I** (2010) Observations on the floral morphology of *Sassafras randaiense* (Lauraceae). *Annals of the Missouri Botanical Garden* 97: 1–10.

181. Lin, C.-R., **Peng, C.-I**, Kono, Y. & Liu, Y. (2010) *Aspidistra obconica*, Asparagaceae [Ruscaceae], a new species from limestone areas in Guangxi, China. *Botanical Studies* 51: 263–268.

182. Gao, Y.-D., Wang, R. J. & **Peng, C.-I** (2010) *Diodia teres* Walt. (Rubiaceae), a newly recorded weed in Fujian. *Taiwania* 55: 177–179.

183. Hsu, T.-W., **Peng, C.-I**, Chiang, T.-Y. & Huang, C.-C. (2010) Three newly naturalized species of the genus *Ludwigia* (Onagraceae) in Taiwan. *Taiwan Journal of Biodiversity* 12: 303–308. (O-17)

184. Hu, W.-H., Chang, C., **Peng, C.-I** & Liaw, S.-I. (2010) *In vitro* flowering and fruiting of *Begonia parvula* H. Lév. & Vaniot. *European Journal of Horticultural Science* 75: 172–176. (*B-32*)

185. Nomura, N., Takaso, T., **Peng, C.-I**, Kono, Y., Oginuma, K., Mitsui, Y. & Setoguchis, H. (2010) Molecular phylogeny and habitat diversification of the genus *Farfugium* (Asteraceae) based on nuclear rDNA and plastid DNA. *Annals of Botany* 106: 467–482. (A-29)

186. Chung, S.-W., Hsu, T.-C. & **Peng, C.-I** (2010) *Phacellanthus* (Orobanchaceae), a newly recorded genus in Taiwan. *Botanical Studies* 51: 531–536.

187. Nakamura, K., Denda, T., Kokubugata, G., Suwa, R., Yang, T. Y. A., **Peng, C.-I** & Yokota, M. (2010) Phylogeography of *Ophiorrhiza japonica* (Rubiaceae) in continental islands, the

Ryukyu Archipelago, Japan. *Journal of Biogeography* 37: 1907–1918.

188. 邵廣昭, 彭鏡毅 & 吳文哲 (eds.) (2010) *臺灣物種名錄2010*. 農委會林務局, 臺北市, 840 pp.

189. Thiele, K. R., Funk, V. A., Iwatsuki, K., Morat, P., **Peng, C.-I**, Raven, P. H., Sarukhan, J. & Seberg, O. (2011) The controversy over the retypification of *Acacia* Mill. with an Australian type: A pragmatic view. *Taxon* 60: 194–198.

190. Hsu, T.-W., Kono, Y., Chiang, T.-Y. & **Peng, C.-I** (2011) *Ypsilandra* (Melanthiaceae; Liliaceae sensu lato), a new generic record for Taiwan. *Botanical Studies* 52: 99–104.

191. Hsu, T.-W., Chiang, T.-Y. & **Peng, C.-I** (2011) *Lobelia cliffortiana* L. (Campanulaceae), a newly naturalized plant in Taiwan. *Taiwan Journal of Biodiversity* 13: 93–96.

192. Kokubugata, G., Nakamura, K., Hirayama, Y., **Peng, C.-I** & Yokota, M. (2011) Taxonomic reconsideration and phylogeographic implication for *Nertera yamashitae* (Rubiaceae). *Bulletin of the National Museum of Nature and Science. Series B, Botany* 37: 9–22.

193. Jung, M.-J., Ku, S.-M. & **Peng, C.-I** (2011) *Oldenlandiopsis* Terell. & W. H. Lewis (Rubiaceae), a newly recorded genus in Taiwan. *Taiwania* 56: 58–61.

194. Kokubugata, G., Hirayama, Y., **Peng, C.-I**, Yokota, M. & Moller, M. (2011) Phytogeographic aspects of *Lysionotus pauciflorus* sensu lato (Gesneriaceae) in the China, Japan and Taiwan regions: phylogenetic and morphological relationships and taxonomic consequences. *Plant Systematics and Evolution* 292: 177–188.

195. Chang, K.-C., Wang, C.-C., Deng, S.-L., Kono, Y., Lu, F.-Y. & **Peng, C.-I** (2011) *Cotoneaster rosiflorus* (Rosaceae), a new species from Taiwan. *Botanical Studies* 52: 211–218.

196. Hsu, T.-W., Chiang, T.-Y. & **Peng, C.-I** (2011) *Heterosmilax septemnervia* F. T. Wang & Tang (Smilacaceae), a newly recorded plant to Taiwan. *Taiwan Journal of Biodiversity* 13: 179–182.

197. Hughes, M., Rubite, R. R., Kono, Y. & **Peng, C.-I** (2011) *Begonia blancii* (sect. *Diploclinium*, Begoniaceae), a new species endemic to the Philippine island of Palawan. *Botanical Studies* 52: 203–209. (*B-33*)

198. Liu, Y., Kono, Y., Lin, C.-R., Xu, W.-B. & **Peng, C.-I** (2011) *Aspidistra erecta* (Asparagaceae), a new species from limestone areas in Guangxi, China. *Botanical Studies* 52: 367–373.

199. 彭鏡毅. (2011) *植物百科圖典*. 貓頭鷹出版社, 台北市, 167 pp. [**Peng, C.-I** (2011) *Botanical Glossary Illustrated*. Owl Publishing House Co., Ltd., Taipei, 167 pp.]

200. Ho, T.-C., **Peng, C.-I**, Chiang, T.-Y. & Hsu, T.-W. (2011) A taxonomical study of the genus *Medicago* (Fabaceae) from Mt. Hohuan of Taiwan. *Taiwan Journal of Biodiversity* 13: 333–344.

201. Chung, K.-F., Lin, T.-T., Tsai, Y.-S., Lin, S.-T. & **Peng, C.-I** (2011) Isolation and characterization of microsatellite loci in *Sassafras randaiense* (Lauraceae). *American Journal of Botany* 98: e326–e329.

202. 彭鏡毅, 黃建益 & 呂文賓. (2011) *鴛鴦湖自然保留區植物解說手冊*. 行政院國軍退除

役輔導委員會榮民森林保育事業管理處，宜蘭市, 268 pp. [**Peng, C.-I**, Huang, C.-I. & Leu, W.-P. (2011) *Guide to Plants of Yuanyang Lake Nature Reserve*. Forest Conservation and Management Administration, Veterans Affairs Council, Ilan, 268 pp.]

203. Xu, W.-B., Pan, B., Liu, Y., **Peng, C.-I** & Chung, K.-F. (2012) Two new species, *Primulina multifida* and *P. pseudomollifolia* (Gesneriaceae), from karst caves in Guangxi, China. *Botanical Studies* 53: 165–175.

204. Nakamura, K., Denda, T., Kokubugata, G., Forster, P. I., Wilson, G., **Peng, C.-I** & Yokota, M. (2012) Molecular phylogeography reveals an antitropical distribution and local diversification of *Solenogyne* (Asteraceae) in the Ryukyu Archipelago of Japan and Australia. *Biological Journal of the Linnean Society* 105: 197–217. (A-30)

205. Nakamura, K., Huang, C.-J., Rubite, R. R., Leong, W.-C., Kono, Y., Yang, H.-A. & **Peng, C.-I** (2012) Isolation of compound microsatellite markers in *Begonia fenicis* (Begoniaceae) endemic to East and Southeast Asia islands. *American Journal of Botany* 99: e20–e23. (*B-34*)

206. 彭鏡毅. (2012) *植物百科圖典, 第二版*. 貓頭鷹出版社，台北市, 175 pp. [**Peng, C.-I** (2011) *Botanical Glossary Illustrated, 2nd edition*. Owl Publishing House Co., Ltd., Taipei, 175 pp.]

207. Sheue, C.-R., Pao, S.-H., Chien, L.-F., Chesson, P. & **Peng, C.-I** (2012) Natural foliar variegation without costs? The case of *Begonia*. *Annals of Botany* 109: 1065–1074. (*B-35*)

208. Tseng, Y.-H., Chao, C.-T., Liou, C.-Y. & **Peng, C.-I** (2012) *Flaveria linearis* Lag. (Asteraceae), a newly naturalized plant in Taiwan. *Quarterly Journal of Forest Research* 34: 63–70. (A-31)

209. Kono, Y., Chung, M.-C. & **Peng, C.-I** (2012) Identification of genome constitutions in *Begonia ×chungii* and its putative parents, *B. longifolia* and *B. palmata*, by genomic in situ hybridization (GISH). *Plant Science* 185-186: 156–160. (*B-36*)

210. **Peng, C.-I**, Ku, S.-M., Kono, Y. & Liu, Y. (2012) *Begonia chongzuoensis* (sect. *Coelocentrum*, Begoniaceae), a new calciphile from Guangxi, China. *Botanical Studies* 53: 285–292. (*B-37*)

211. Nakamura, K., Chung, K.-F., Huang, C.-J., Kono, Y., Kokubugata, G. & **Peng, C.-I** (2012) Extreme habitats that emerged in the Pleistocene triggered divergence of weedy *Youngia* (Asteraceae) in Taiwan. *Molecular Phylogenetics and Evolution* 63: 486–499. (A-32)

212. **Peng, C.-I**, Kono, Y., Chen, C.-J., Hsu, T.-C. & Chung, S.-W. (2012) *Pouzolzia taiwaniana* (Urticaceae), a new species from Taiwan. *Botanical Studies* 53: 387–392.

213. 胡維新，**彭鏡毅** & 廖松淵. (2012) 小葉秋海棠葉片培養不定芽再生. *植物種苗* 14: 21–32. [Hu, W.-H., **Peng, C.-I** & Liaw, S.-I. (2012) Adventitious bud regeneration from lamina culture of *Begonia parvula* H. Lev. & Vaniot. *Seed & Nursery (Taiwan)* 14: 21–32.] (*B-38*)

214. Kono, Y., Chung, K.-F., Chen, C.-H., Hoshi, Y., Setoguchi, H., Chou, C.-H., Oginuma, K. & **Peng, C.-I** (2012) Intraspecific karyotypic polymorphism is highly concordant with allozyme variation in *Lysimachia mauritiana* (Primulaceae: Myrsinoideae) in Taiwan:

implications for the colonization history and dispersal patterns of coastal plants. *Annals of Botany* 110: 1119–1135.

215. 彭鏡毅，穆家宏，黃建益 & 胡嘉穎. (2012) 中央研究院植物數位典藏之現況與未來展望. *In*: 邵廣昭，謝長富 & 何恭算 (eds.) *台灣生物多樣性與地質資料庫.* 中央研究院生物多樣性研究中心，台北市, pp. 19–25. [**Peng, C.-I**, Mu, C.-H., Huang, C.-I. & Hu, C.-Y. (2012) The present and future of the digital archive of plant database, Academia Sinica. *In*: Shao, K.-T., Hsieh, C.-F. & Ho, K.-S. (eds.) *Biodiversity and Geology Databases of Taiwan**. Biodiversity Research Center, Academia Sinica, Taipei, pp. 19–25.]

216. Wanntorp, L., De Craene, L. R., **Peng, C.-I** & Anderberg, A. A. (2012) Floral ontogeny and morphology of *Stimpsonia* and *Ardisiandra*, two aberrant genera of the *Primuloid* clade of Ericales. *International Journal of Plant Sciences* 173: 1023–1035.

217. Liu, Y., Xu, W.-B., Huang, Y.-S., **Peng, C.-I** & Chung, K.-F. (2012) *Oreocharis dayaoshanioides*, a rare new species of Gesneriaceae from eastern Guangxi, China. *Botanical Studies* 53: 393–399.

218. Chung, K.-F., Huang, H.-Y., **Peng, C.-I** & Xu, W.-B. (2013) *Primulina mabaensis* (Gesneriaceae), a new species from a limestone cave of northern Guangdong, China. *Phytotaxa* 92: 40–48.

219. Nakamura, K., Kono, Y., Huang, C.-J., Chung, K.-F. & **Peng, C.-I** (2013) Correction of confusions regarding the identity and synonymy of *Youngia* (Asteraceae: Tribe Cichorieae) in Taiwan. *Systematic Botany* 38: 507–516. (A-33)

220. Xu, W.-B., Liu, Y., Kono, Y., Chang, H., **Peng, C.-I** & Chung, K.-F. (2013) *Primulina cardaminifolia* (Gesneriaceae), a rare new species from limestone areas in Guangxi, China. *Botanical Studies* 24: e19.

221. Oguri, E., Sugawara, T., **Peng, C.-I**, Yang, T.-Y. A. & Murakami, N. (2013) Geographical origin and sexual-system evolution of the androdioecious plant *Gynochthodes boninensis* (Rubiaceae), endemic to the Bonin Islands, Japan. *Molecular Phylogenetics and Evolution* 68: 699–708.

222. Rubite, R. R., Hughes, M., Alejandro, G. & **Peng, C.-I** (2013) Recircumscription of *Begonia* sect. *Baryandra* (Begoniaceae): evidence from molecular data. *Botanical Studies* 54: e38. (*B-39*)

223. Lin, C.-R., Liu, Y., Nong, D.-X., Kono, Y. & **Peng, C.-I** (2013) *Aspidistra crassifila* (Asparagaceae), a new species from Guangxi, China. *Botanical Studies* 54: e43.

224. **Peng, C.-I**, Yang, H.-A., Kono, Y., Chung, K.-F., Huang, Y.-S., Wu, W.-H. & Liu, Y. (2013) Novelties in *Begonia* sect. *Coelocentrum*: *B. longgangensis* and *B. ferox* from limestone areas in Guangxi, China. *Botanical Studies* 54: e44. (*B-40*)

225. 海峽兩岸生態學名詞工作委員會 (ed.) (2013) *海峽兩岸生態學名詞.* 科學出版社，北京, 308 pp. [Editorial Committee of Cross-Strait Ecological Terms (ed.) (2013) *Cross-Strait Ecological Terms**. Science Press, Beijing, pp. 308.*] [Committee Member]

226. Nakamura, K., Rubite, R. R., Kono, Y., Callado, J. R. & **Peng, C.-I** (2013) *Begonia tandangii* (Begoniaceae, section *Baryandra*), a new species from Luzon Island, the

Philippines. *Phytotaxa* 145: 27–37. (*B-41*)

227. Nakamura, K., Rubite, R. R., Kokubugata, G., Kono, Y., Yokota, M. & **Peng, C.-I** (2013) First record of the genus *Limonium* (Plumbaginaceae) from the Malesian region. *Phytotaxa* 152: 56–58.

228. Chung, K.-F., Leong, W.-C., Rubite, R. R., Repin, R., Kiew, R., Liu, Y. & **Peng, C.-I** (2014) Phylogenetic analyses of *Begonia* sect. *Coelocentrum* and allied limestone species of China shed light on the evolution of Sino-Vietnamese karst flora. *Botanical Studies* 55: e1. (*B-42*)

229. **Peng, C.-I**, Wang, H., Kono, Y. & Yang, H.-A. (2014) *Begonia wui-senioris* (sect. *Platycentrum*, Begoniaceae), a new species from Myanmar. *Botanical Studies* 55: e13. (*B-43*)

230. **Peng, C.-I**, Jin, X.-H., Ku, S.-M., Kono, Y., Huang, H.-Y. & Yang, H.-A. (2014) *Begonia wuzhishanensis* (sect. *Diploclinium*, Begoniaceae), a new species from Hainan Island, China. *Botanical Studies* 55: e24. (*B-44*)

231. Nakamura, K., Kokubugata, G., Rubite, R. R., Huang, C.-J., Kono, Y., Yang, H.-A., Lopez-Feliciano, A., Labuguen, M. L., Yokota, M. & **Peng, C.-I** (2014) *In situ* glacial survival at the northern limit of tropical insular Asia by a lowland herb *Begonia fenicis* (Begoniaceae). *Botanical Journal of the Linnean Society* 174: 305–325. (*B-45*)

232. Nakamura, K., Ma, C.-J., Huang, C.-J., Ho, M.-J., Yang, H.-A. & **Peng, C.-I** (2014) Development and characterization of microsatellite markers in the coffee-family herb *Ophiorrhiza japonica* (Rubiaceae). *Conservation Genetics Resources* 6: 447–449.

233. **Peng, C.-I**, Ku, S.-M., Yang, H.-A., Leong, W.-C., Liu, Y., Nguyen, T. H., Kono, Y. & Chung, K.-F. (2014) Two new species of *Begonia* sect. *Coelocentrum*, *B. guixiensis* and *B. longa*, from Sino-Vietnamese limestone karsts. *Botanical Studies* 55: e52. (*B-46*)

234. Ding, B., Nakamura, K., Kono, Y., Ho, M.-J. & **Peng, C.-I** (2014) *Begonia jinyunensis* (Begoniaceae, section *Platycentrum*), a new palmately compound leaved species from Chongqin, China. *Botanical Studies* 55: e62. (*B-47*)

235. Nakamura, K., Ho, M.-J., Ma, C.-J., Yang, H.-A. & **Peng, C.-I** (2014) Development of EST-derived microsatellite markers in *Ophiorrhiza pumila* (Rubiaceae) and their application in congeners. *Conservation Genetics Resources* 6: 649–651.

236. Nakamura, K., Chung, S.-W., Kono, Y., Ho, M.-J., Hsu, T.-C. & **Peng, C.-I** (2014) *Ixeridium calcicola* (Compositae), a new limestone endemic from Taiwan, with notes on its atypical basic chromosome number, phylogenetic affinities, and a limestone refugium hypothesis. *Plos ONE* 9: e10979. (A-34)

237. 彭鏡毅. (2014) 陸域植物：異域小島，草木殊勝. *In*: 邵廣昭 & 林幸助 (eds.) *南疆沃海--南沙太平島生物多樣性*. 內政部營建署, 台北市, pp. 27–42. [**Peng, C.-I** (2014) Terrestrial plants: Exotic faraway island, astounding and unique. *In*: Shao, K.-T. & Lin, H.-J. (ed.) *A Frontier in the South China Sea: Biodiversity of Taiping Island, Nansha Islands*. Construction and Planning Agency, Minister of the Interior, Taipei, pp. 27–42.]

238. Lin, C.-W., Chung, S.-W. & **Peng, C.-I** (2014) *Begonia hosensis* (sect. *Reichenheimia*,

Begoniaceae), a new species from Sarawak, Malaysia. *Taiwania* 59: 326–330. (*B-48*)

239. Lin, C.-W. & **Peng, C.-I** (2014) *Begonia natunaensis* (sect. *Reichenheimia*, Begoniaceae), a new species from Natuna Island, Indonesia. *Taiwania* 59: 368–373. (*B-49*)

240. Lin, C.-W., Chung, S.-W. & **Peng, C.-I** (2014) Three new species of *Begonia* (sect. *Petermannia*, Begoniaceae) from Sarawak, Borneo. *Phytotaxa* 191: 129–140. (*B-50*)

241. Lin, C.-W., Chung, S.-W. & **Peng, C.-I** (2015) *Begonia baik* and *B. padawanensis* spp. nov. (Begoniaceae) from sandstone areas in Sarawak, Malaysia. *Nordic Journal of Botany* 33: 555–561. (*B-51*)

242. Nakamura, K., Denda, T., Kokubugata, G., Huang, C.-J., **Peng, C.-I** & Yokota, M. (2015) Phylogeny and biogeography of the *Viola iwagawae-tashiroi* species complex (Violaceae, section Plagiostigma) endemic to the Ryukyu Archipelago, Japan. *Plant Systematics and Evolution* 301: 337–351.

243. 彭鏡毅. (2015) *植物百科圖典, 第三版.* 貓頭鷹出版社, 台北市, 191 pp. [**Peng, C.-I** (2011) *Botanical Glossary Illustrated, 3rd edition*. Owl Publishing House Co., Ltd., Taipei, 191 pp.]

244. Nakamura, K., Wang, Y.-F., Ho, M.-J., Chung, K.-F. & **Peng, C.-I** (2015) New distribution record of *Begonia grandis* (Begoniaceae, section *Diploclinium*) from Taiwan, with subspecies assignment based on morphology and molecular phylogeny. *Taiwania* 60: 49–53. (*B-52*)

245. Moonlight, P. W., Richardson, J. E., Tebbitt, M. C., Thomas, D. C., Hollands, R., **Peng, C.-I** & Hughes, M. (2015) Continental-scale diversification patterns in a megadiverse genus: the biogeography of Neotropical Begonia. *Journal of Biogeography* 42: 1137–1149. (*B-53*)

246. **Peng, C.-I**, Lin, C.-W., Repin, R., Kono, Y., Leong, W.-C. & Chung, K.-F. (2015) Two new species of *Begonia*, *B. moneta* and *B. peridoticola* (Begoniaceae) from Sabah, Malaysia. *Botanical Studies* 56: e7. (*B-54*)

247. 彭鏡毅. (2015) *植物學中英百科圖典.* 貓頭鷹出版社, 台北市, 351 pp. [**Peng, C.-I** (2015) The Chinese-English Illustrated Botanical Glossary. Owl Publishing House Co., Ltd., Taipei, 351 pp.]

248. Hughes, M., Rubite, R. R., Blanc, P., Chung, K.-F. & **Peng, C.-I** (2015) The Miocene to Pleistocene colonization of the Philippine archipelago by *Begonia* sect. *Baryandra* (Begoniaceae). *American Journal of Botany* 102: 695–706. (*B-55*)

249. **Peng, C.-I**, Lin, C.-W., Yang, H.-A., Kono, Y. & Nguyen, H. Q. (2015) Six new species of *Begonia* (Begoniaceae) from limestone areas in Northern Vietnam. *Botanical Studies* 56: e9. (*B-56*)

250. Watanabe, K., Yang, T. Y. A., Nishihara, C., Huang, T. L., Nakamura, K., Peng, C. I. & Sugawara, T. (2015) Distyly and floral morphology of *Psychotria cephalophora* (Rubiaceae) on the oceanic Lanyu (Orchid) Island, Taiwan. *Botanical Studies* 56: e10.

251. Lin, W.-C., Wen, C.-C., Chen, Y.-H., Hsiao, P.-W., Liao, J.-W., **Peng, C.-I** & Yang, N.-S. (2015) Integrative approach to analyze biodiversity and anti-Inflammatory bioactivity of *Wedelia* medicinal plants. *PLoS ONE* 10: e012906. (A-35)

252. Rubite, R. R., Hughes, M., Blanc, P., Chung, K.-F., Yang, H.-A., Kono, Y., Alejandro, G. J. D., De Layola, L. B., Virata, A. G. N. & **Peng, C.-I** (2015) Three new species of *Begonia* endemic to the Puerto Princesa Subterranean River National Park, Palawan. *Botanical Studies* 56: e19. (*B-57*)
253. **Peng, C.-I**, Yang, H.-A., Kono, Y., Jung, M.-J. & Nguyen, T. H. (2015) Four new species of *Begonia* (Begoniaceae) from Vietnam: *B. abbreviata*, *B. calciphila*, *B. sphenantheroides* and *B. tamdaoensis*. *Phytotaxa* 222: 83–99. (*B-58*)
254. Leong, W.-C., Deng, T., Sun, H., **Peng, C.-I** & Chung, K.-F. (2015) *Begonia difformis comb. & stat. nov.* (Sect. *Platycentrum*, Begoniaceae), a new species segregated from *B. palmata* D. Don. *Phytotaxa* 227: 83–91. (*B-59*)
255. Tanaka, N. & **Peng, C.-I** (2016) *Begonia togashii* (Begoniaceae: Sect. *Platycentrum*), a new species from central Myanmar. *Acta Phytotaxonomica et Geobotanica* 67: 191–197. (*B-60*)
256. **Peng, C.-I**, Rubite, R. R. & Lin, C.-W. (2017) *Begonia polyclada* (sect. *Petermannia*, Begoniaceae), a gracile new species from Luzon, Philippines. *Phytotaxa* 296: 93–97. (*B-61*)
257. Lin, C.-W. & **Peng, C.-I** (2017) Three new species of *Begonia* (Begoniaceae) from limestone hills in southwestern Sarawak, Borneo. *Taiwania* 62: 105–115. (*B-62*)
258. Tseng, Y.-H., Kim, Y.-D., **Peng, C.-I**, Htwe, K. M., Cho, S.-H., Kono, Y. & Chung, K.-F. (2017) *Begonia myanmarica* (Begoniaceae), a new species from Myanmar, and molecular phylogenetics of *Begonia* sect. *Monopteron*. *Botanical Studies* 58: e21. (*B-63*)
259. Yoichi, W., Jin, X.-F., **Peng, C.-I**, Tamaki, I. & Tomaru, N. (2017) Contrasting diversification history between insular and continental species of three-leaved azaleas (*Rhododendron* sect. *Brachycalyx*) in East Asia. *Journal of Biogeography* 44: 1065–1076.
260. Tseng, Y.-H., Huang, H.-Y., Xu, W.-B., Yang, H.-A., Liu, Y., **Peng, C.-I** & Chung, K.-F. (2017) Development and characterization of EST-SSR markers for *Begonia luzhaiensis* (Begoniaceae). *Applications in Plant Sciences* 5: e1700024. (*B-64*)
261. Lin, C.-W., Thomas, D. C., Ardi, W. H. & **Peng, C.-I** (2017) *Begonia ignita* (sect. *Petermannia*, Begoniaceae), a new species with orange flowers from Sulawesi, Indonesia. *Gardens' Bulletin Singapore* 69: 89–95. (*B-65*)
262. **Peng, C.-I**, Rubite, R. R., Lin, C.-W., Hughes, M., Kono, Y. & Chung, K.-F. (2017) Three new species of *Begonia* sect. *Baryandra* from Panay Island, Philippines. *Botanical Studies* 58: e28. (*B-66*)
263. Lin, C.-W., Chung, S.-W. & **Peng, C.-I** (2017) Eleven new species of *Begonia* (Begoniaceae) from Sarawak, Borneo. *Taiwania* 62: 219–251. (*B-67*)
264. Qin, Y.-H., Liang, Y.-Y., Xu, W.-B., Lin, C.-W. & **Peng, C.-I** (2017) *Begonia ufoides* (sect. *Coelocentrum*, Begoniaceae), a new species from limestone areas in central Guangxi, China. *Phytotaxa* 316: 279–284. (*B-68*)
265. **Peng, C.-I**, Lin, C.-W. & Phutthai, T. (2017) *Begonia fulgurata* (sect. *Diploclinium*, Begoniaceae), a new species from northern Thailand. *Blumea* 62: 163–167. (*B-69*)
266. Averyanov, L. V., Tanaka, N., Son, H. T., Nguyen, K. S., Maisak, T. V., Nguyen, T. H. & **Peng, C.-I** (2018) *Tupistra cardinalis* (Asparagaceae), a new species from limestone areas

in northern Vietnam. *Phytotaxa* 334: 60–64.

267. Lý, N.-S., **Peng, C.-I** & Hughes, M. (2018) *Begonia lecongkietii* (sect. *Petermannia*), a new species from Mount Dau, South Central Coast Region, Vietnam *Edinburgh Journal of Botany* 75: 167–172. (*B-70*)

268. Pao, S.-H., Tsai, P.-Y., **Peng, C.-I**, Chen, P.-J., Tsai, C.-C., Yang, E.-C., Shih, M.-C., Chen, J., Yang, J.-Y., Chesson, P. & Sheue, C.-R. (2018) Lamelloplasts and minichloroplasts in Begoniaceae: iridescence and photosynthetic functioning. *Journal of Plant Research* 131: 655–670. (*B-71*)

269. Moonlight, P. W., Ardi, W. H., Padilla, L. A., Chung, K.-F., Fuller, D., Deden Girmansyah, Hollands, R., Adolfo Jara-Muñoz, Kiew, R., Wai-Chao Leong, Liu, Y., Mahardika, A., Marasinghe, L. D. K., O'Connor, M., **Peng, C.-I**, Pérez, Á . J., Phutthai, T., Pullan, M., Rajbhandary, S., Reynel, C., Rubite, R. R., Sang, J., Scherberich, D., Shui, Y.-M., Tebbitt, M. C., Thomas, D. C., Wilson, H. P., Zaini, N. H. & Hughes, M. (2018) Dividing and conquering the fastest growing genus: Towards a natural sectional classification of the mega-diverse genus *Begonia* (Begoniaceae). *Taxon* 67: 267–323. (*B-72*)

270. Rubite, R. R., **Peng, C.-I**, Chung, K.-F., Lin, C.-W., Evangelista, L. T., Tandang, D. N., Callado, J. R. C. & Hughes, M. (2018) Three new species of *Begonia* (section *Baryandra*, Begoniaceae) from Luzon Island, the Philippines. *Phytotaxa* 347: 201–212. (*B-73*)

271. Hughes, M., **Peng, C.-I**, Lin, C.-W., Rubite, R. R., Blanc, P. & Chung, K.-F. (2018) Chloroplast and nuclear DNA exchanges among *Begonia* sect. *Baryandra* species (Begoniaceae) from Palawan Island, Philippines, and descriptions of five new species. *PLoS ONE* 13: e0194877. (*B-74*)

272. Li, H.-Z., Guan, K.-Y., Lin, C.-W. & **Peng, C.-I** (2018) *Begonia qingchengshanensis* (sect. *Reichenheimia*, Begoniaceae), a new species from Sichuan, China. *Phytotaxa* 349: 197–200. (*B-75*)

273. Hughes, M. & **Peng, C.-I** (eds.) (2018) *Asian Begonia: 300 Species Portraits*. KBCC Press & RBGE, Taipei, Taiwan & Edinburgh, Scotland, 353 pp. (*B-76*)

274. Jung, M.-J., Lu, W.-P., **Peng, C.-I** & Tseng, Y.-H. (2018) *Pyrrhopappus* DC. (Asteraceae), a new-recorded genus and its naturalized species to the Flora of Taiwan. *Quarterly Journal of Forest Research* 40: 185–190. (A-36)

275. 彭鏡毅 (2018) *為愛走天涯：踏覓秋海棠*. 麥浩斯出版, 台北市, 291 pp. [**Peng, C.-I** (2018) *Endless Trekking in Search of Begonia*. My House Publishing Co., Ltd., Taipei, 291 pp.] (*B-77*)

276. **Peng, C.-I**, Truong, N.-D., Nguyen, D.-D. & Lin, C.-W. (2018) *Begonia austrovietnamica* (sect. *Alicida*, Begoniaceae), a handsome new species from South Vietnam. *Phytotaxa* 381: 95–99. (*B-78*)

277. Kono, Y., **Peng, C.-I**, Hoshi, Y., Yokota, M., Setoguchi, H., Lum, S. K. Y. & Oginuma, K. (2019) Intraspecific karyotype polymorphism and chromosomal evolution of *Lysimachia mauritiana* (Primulaceae) in the Ryukyu Archipelago of Japan and Taiwan. *Cytologia* 84: 93–103.

278. Lin, C.-W. & **Peng, C.-I** (2019) Five new species of *Begonia* (sect. *Petermannia*, Begoniaceae) from western Sarawak, Borneo. Taiwania 64: 124–138. (*B-79*)

279. Liu, S.-H., Tseng, Y.-H., Zure, D., Rubite, R. R., Balangcod, T. D., **Peng, C.-I** & Chung, K.-F. (2019) *Begonia balangcodiae sp. nov.* from northern Luzon, the Philippines and its natural hybrid with *B. crispipila*, *B.* × *kapangan nothosp. nov. Phytotaxa* 407: 5–21. (*B-80*)

280. Tseng, Y.-H., Huang, H.-Y., Xu, W.-B., Yang, H.-A., **Peng, C.-I**, Liu, Y. & Chung, K.-F. (2019) Phylogeography of *Begonia luzhaiensis* suggests both natural and anthropogenic causes for the marked population genetic structure. *Botanical Studies* 60: e20. (*B-81*)

281. Liu, S.-H., Yang, H.-A, Kono, Y., Hoch, P. C., Barber, J. C., **Peng, C.-I** & Chung, K.-F. (2020) Disentangling reticulated evolution of North Temperate Haplostemonous *Ludwigia* (Onagraceae). *Annals of the Missouri Botanical Garden* (in press) (O-18)
