# Supplementary material for: In memoriam Ching-I Peng (1950–2018)—an outstanding scientist and mentor with a remarkable legacy
Source: Bot Stud. 2020 Apr 25;61:14. doi: 10.1186/s40529-020-00291-5 (PMC7182648; doi:10.1186/s40529-020-00291-5)
Supplement: Supplementary file 3 — Additional file 3. New taxa and names (121) published by Ching-I Peng. [file 40529_2020_291_MOESM3_ESM.pdf]

**Additional file 3. New taxa and names (121) published by Ching-I Peng.** See Additional file 2 (superscripts following the Chinese plant names) for relevant reference.

**Asteraceae** (12)

1. *Blumea linearis* C.I Peng & W.P.Leu 狹葉艾納香 [74]
2. *Eupatorium clematideum* var. *gracillimum* (Hayata) C.I Peng & S.W.Chung 高士佛澤蘭 [72]
3. *Eupatorium hualienense* C.H.Ou, S.W.Chung & C.I Peng 花蓮澤蘭 [72]
4. *Ixeridium calcicola* C.I Peng, S.W.Chung & T.C.Hsu 喜鈣刀傷草 [236]
5. *Parasenecio hwangshanicus* (Ling) C.I Peng & S.W.Chung 黃山蟹甲草 [72]
6. *Parasenecio monanthus* (Diels) C.I Peng & S.W.Chung 玉山蟹甲草 [72]
7. *Parasenecio morrisonensis* Ying Liu, C.I Peng & Q.E.Yang 玉山蟹甲草 [143]
8. *Parasenecio nokoensis* (Masam. & Suzuki) C.I Peng & S.W.Chung 能高蟹甲草 [72]
9. *Pterocypsela* × *mansuensis* (Hayata) C.I Peng 恆春山苦蕒 [72]
10. *Senecio kuanshanensis* C.I Peng & S.W.Chung 關山千里光 [100]
11. *Senecio tarokoensis* C.I Peng 太魯閣千里光 [74]
12. *Youngia japonica* subsp. *monticola* Koh Nakam. & C.I Peng 山間黃鵪菜 [219]

**Begoniaceae** (98)

13. *B. abbreviata* C.I Peng 短序秋海棠 [VN, 244]
14. *B. aiensis* C.W.Lin & C.I Peng 艾河秋海棠 [MY, 263]
15. *B. amidalae* C.W.Lin & C.I Peng 艾米達拉秋海棠 [MY, 240]
16. *B. anserina* C.W.Lin & C.I Peng 雁行秋海棠 [MY, 240]
17. *B. arachnoidea* C.I Peng, Yan Liu & S.M.Ku 蛛網脈秋海棠 [CN, 160]
18. *B. aurantiflora* C.I Peng, Yan Liu & S.M.Ku 橙花側膜秋海棠 [CN, 152]
19. *B. austrotaiwanensis* Y.K.Chen & C.I Peng 南台灣秋海棠 [TW, 37]
20. *B. austrovietnamica* C.I Peng, C.W.Lin, D.D.Nguyen & N.D.Truong 南越秋海棠 [VN, 276]
21. *B. baik* C.W.Lin & C.I Peng 雪絨苞秋海棠 [MY, 241]
22. *B. bamaensis* Yan Liu & C.I Peng 巴馬秋海棠 [CN, 149]
23. *B. blancii* M.Hughes & C.I Peng 匙葉秋海棠 [PH, 197]
24. *B. bouffordii* C.I Peng 九九峰秋海棠 [TW, 123]
25. *B.* × *breviscapa* C.I Peng, Yan Liu & S.M.Ku 短葶秋海棠 [CN, 176]
26. *B. calciphila* C.I Peng 喜鈣秋海棠 [VN, 244]
27. *B. caobangensis* C.I Peng & C.W.Lin 高平秋海棠 [VN, 249]
28. *B. chongzuoensis* Yan Liu, S.M.Ku & C.I Peng 崇左秋海棠 [CN, 210]
29. *B.* × *chungii* C.I Peng & S.M.Ku 鍾氏秋海棠 [TW, 169]
30. *B. chuyunshanensis* C.I Peng & Y.K.Chen 出雲山秋海棠 [TW, 123]
31. *B. circularis* C.I Peng & C.W.Lin 環紋秋海棠 [VN, 249]
32. *B. coptidifolia* H.G.Ye, F.G.Wang, Y.S.Ye & C.I Peng 陽春秋海棠 [CN, 114]
33. *B. crocea* C.I Peng 橙花秋海棠 [CN, 130]
34. *B. culasiensis* C.I Peng, Rubite, C.W.Lin & K.F.Chung 庫拉西秋海棠 [PH, 262]
35. *B. curvicarpa* S.M.Ku, C.I Peng & Yan Liu 彎果秋海棠 [CN, 117]
36. *B. darthvaderiana* C.W.Lin & C.I Peng 黑武士秋海棠 [MY, 240]

37. *B. debaoensis* C.I Peng, Yan Liu & S.M.Ku 德保秋海棠 [CN, 133]
38. *B. difformis* (Irmsch.) W.C.Leong, C.I Peng & K.F.Chung 刺毛紅孩兒 [CN, 254]
39. *B. dinosauria* C.W.Lin & C.I Peng 恐龍皮秋海棠 [MY, 263]
40. *B. droseroides* C.I Peng, Rubite & C.W.Lin 毛氈苔狀秋海棠 [PH, 270]
41. *B. elnidoensis* C.I Peng, Rubite & C.W.Lin 艾尼朵秋海棠 [PH, 271]
42. *B. fangii* Y.M.Shui & C.I Peng 方氏秋海棠 [CN, 119]
43. *B. felis* C.W.Lin & C.I Peng 貓城秋海棠 [MY, 257]
44. *B. ferox* C.I Peng & Yan Liu 黑峰秋海棠 [CN, 224]
45. *B. fulgurata* C.I Peng, C.W.Lin & Phutthai 電光秋海棠 [TH, 265]
46. *B. gabaldonensis* Rubite, C.I Peng & C.W.Lin 蓋伯頓秋海棠 [PH, 270]
47. *B. gironellae* C.I Peng, Rubite & C.W.Lin 貝絲秋海棠 [PH, 271]
48. *B. handelii* Irmsch. var. *rubropilosa* (S.H.Huang & Y.M.Shui) C.I Peng 紅毛香花秋海棠 [CN]
49. *B. hirsuticarpa* C.W.Lin & C.I Peng 毛果秋海棠 [MY, 263]
50. *B. hosensis* C.W.Lin & C.I Peng 赫斯山秋海棠 [MY, 238]
51. *B. hughesii* Rubite & C.I Peng 休斯氏秋海棠 [PH, 252]
52. *B. ignita* C.W.Lin & C.I Peng[ID, 261]
53. *B. iridifolia* C.W.Lin & C.I Peng 虹彩葉秋海棠 [MY, 263]
54. *B. jinyunensis* C.I Peng, Bo Ding & Qian Wang 縉雲秋海棠 [CN, 234]
55. *B. kuchingensis* C.W.Lin & C.I Peng 古晉秋海棠 [MY, 257]
56. *B. kui* C.I Peng 麗紋秋海棠 [VN, 140]
57. *B. langsonensis* C.I Peng & C.W.Lin 諒山秋海棠 [VN, 249]
58. *B. lawii* C.W.Lin & C.I Peng 羅氏秋海棠 [MY, 263]
59. *B. lichenora* C.W.Lin & C.I Peng 地衣狀秋海棠 [MY, 263]
60. *B. liuyanii* C.I Peng, S.M.Ku & W.C.Leong 劉演秋海棠 [CN, 122]
61. *B. locii* C.I Peng, C.W.Lin & H.Q.Nguyen 織錦秋海棠 [VN, 249]
62. *B. longgangensis* C.I Peng & Yan Liu 弄崗秋海棠 [CN, 224]
63. *B. luochengensis* S.M.Ku, C.I Peng & Yan Liu 羅城秋海棠 [CN, 117]
64. *B. madulidii* Rubite, C.I Peng & C.W.Lin 馬杜里德秋海棠 [PH, 270]
65. *B. magnicarpa* C.W.Lin & C.I Peng 巨果秋海棠 [MY, 263]
66. *B. melanobullata* C.I Peng & C.W.Lin 越南黑峰秋海棠 [VN, 249]
67. *B. merrilliana* C.I Peng, Rubite, C.W.Lin & K.F.Chung 美林秋海棠 [PH, 262]
68. *B. metallicolor* C.W.Lin & C.I Peng 釉藍秋海棠 [MY, 263]
69. *B. moneta* C.I Peng, Rimi & C.W.Lin 銀幣秋海棠 [MY, 246]
70. *B. montaniformis* C.I Peng, C.W.Lin & H.Q.Nguyen 山峰秋海棠 [VN, 249]
71. *B. myanmarica* C.I Peng & Y.D.Kim[MM, 258]
72. *B. natunaensis* C.W.Lin & C.I Peng 納圖那秋海棠 [ID, 239]
73. *B. ningmingensis* D.Fang, Y.G.Wei & C.I Peng 寧明秋海棠 [CN, 131]
74. *B. ningmingensis* var. *bella* D.Fang, Y.G.Wei & C.I Peng 麗葉秋海棠 [CN, 131]
75. *B. nix* C.W.Lin & C.I Peng 飄雪秋海棠 [MY, 263]
76. *B. opaca* C.W.Lin & C.I Peng 紺碧秋海棠 [MY, 278]

77. *B. padawanensis* C.W.Lin & C.I Peng 巴達旺秋海棠 [MY, 241]
78. *B. paracauliflora* Rimi, C.I Peng & S.M.Ku[MY] in Rupin et al. (2015)
79. *B. peridoticola* Rimi, C.I Peng & C.W.Lin 橄欖岩秋海棠 [MY, 246]
80. *B. picturata* Yan Liu, S.M.Ku & C.I Peng 一口血秋海棠 [CN, 125]
81. *B. pinglinensis* C.I Peng 坪林秋海棠 [TW, 123]
82. *B. polyclada* C.I Peng, C.W.Lin & Rubite 多枝秋海棠 [PH, 256]
83. *B. pseudodaxinensis* S.M.Ku, Yan Liu & C.I Peng 假大新秋海棠 [CN, 133]
84. *B. pseudoleprosa* C.I Peng, Yan Liu & S.M.Ku 假癩葉秋海棠 [CN, 133]
85. *B. pulvinifera* C.I Peng & Yan Liu 腫柄秋海棠 [CN, 134]
86. *B. qingchengshanensis* H.Z.Li, C.I Peng & C.W.Lin 青城山秋海棠 [CN, 272]
87. *B. quinquealata* C.I Peng, Rubite & C.W.Lin 五翼秋海棠 [PH, 271]
88. *B. ravenii* C.I Peng & Y.K.Chen 岩生秋海棠 [TW, 29]
89. *B. retinervia* D.Fang, D.H.Qin & C.I Peng 突脈秋海棠 [CN, 131]
90. *B. semiparietalis* Yan Liu, S.M.Ku & C.I Peng 半側膜秋海棠 [CN, 133]
91. *B. serianensis* C.W.Lin & C.I Peng 西連秋海棠 [MY, 257]
92. *B. sphenantheroides* C.I Peng 高莖秋海棠 [VN, 244]
93. *B. subcoriacea* C.I Peng, Yan Liu & S.M.Ku 近革葉秋海棠 [CN, 160]
94. *B. superciliaris* C.W.Lin & C.I Peng 畫眉秋海棠 [MY, 263]
95. *B. sykakiengii* Rubite, C.I Peng, C.W.Lin & K.F.Chung 施氏秋海棠 [PH, 262]
96. *B. tabonensis* C.I Peng, Rubite & C.W.Lin 塔邦洞秋海棠 [PH, 271]
97. *B. tagbanua* M.Hughes, C.I Peng & Rubite[PH, 252]
98. *B.* × *taipeiensis* C.I Peng 台北秋海棠 [TW, 82]
99. *B. tamdaoensis* C.I Peng 三島秋海棠 [VN, 244]
100. *B. taraw* C.I Peng, Rubite & M.Hughes[PH, 252]
101. *B. tengchiana* C.I Peng & Y.K.Chen 藤枝秋海棠 [TW, 123]
102. *B. tenuibracteata* C.I Peng, Rubite & C.W.Lin[PH, 271]
103. *B. togashii* Nob.Tanaka & C.I Peng[MM, 255]
104. *B. ufoides* C.I Peng, Y.H.Qin & C.W.Lin 碟葉秋海棠 [CN, 264]
105. *B. vietnamensis* H.Q.Nguyen & C.I Peng 越南秋海棠 [VN, 175]
106. *B. wallacei* C.W.Lin & C.I Peng 華萊士秋海棠 [MY, 263]
107. *B. wui-senioris* C.I Peng 曼德勒秋海棠 [MM, 229]
108. *B. wutaiana* C.I Peng & Y.K.Chen 霧台秋海棠 [TW, 123]
109. *B. wuzhishanensis* C.I Peng, X.H.Jin & S.M.Ku 五指山秋海棠 [CN, 230]
110. *B. zygia* C.W.Lin & C.I Peng 對葉秋海棠 [MY, 278]

**Commelinaceae**

111. *Belosynapsis kawakamii* (Hayata) C.I Peng & Yo J.Chen 川上氏鴨舌疝 [89]

**Asparagaceae**

112. *Aspidistra crassifila* Yan Liu & C.I Peng 粗絲蜘蛛抱蛋 [223]
113. *Aspidistra erecta* Yan Liu & C.I Peng 直立蜘蛛抱蛋 [198]

**Liliaceae**

114. *Tricyrtis ravenii* C.I Peng & Tiang 高山油點草 [147]

**Gentianaceae**

115. *Gentiana bambuseti* T.Y.Hsieh, T.C.Hsu, S.M.Ku & C.I Peng 竹林龍膽 [146]

**Onagraceae**

116. *Ludwigia glandulosa* Walter subsp. *brachycarpa* (Torrey & A.Gray) C.I Peng[15] = *Ludwigia glandulosa* Walter subsp. *brachycarpa* C.I Peng in Hoch & Gandhi (2020)
117. *Ludwigia ravenii* C.I Peng  雷氏水丁香 [10]
118. *Ludwigia* × *taiwanensis* C.I Peng 台灣水龍 [36]

**Primulaceae**

119. *Lysimachia chingshuiensis* C.I Peng & C.M.Hu 清水山過路黃 [73]
120. *Lysimachia ravenii* C.I Peng 大漢山珍珠菜  in Chung (2018)

**Urticaceae**

121. *Pouzolzia taiwaniana* C.I Peng & S.W.Chung 臺灣霧水葛 [212]

Distributions of the 98 *Begonia* species/variety named by Ching-I Peng: [CN]China (29 species), [IN]Indonesia (2 species), [MM]Myanmar (3 species), [MY]Malaysia (25 species), [PH]Philippines (15 species), [TH]Thailand (1), [TW]Taiwan (10), and [VN]Vietnam (13).

## Reference

Chung S-W (2018) Illustrated Flora of Taiwan, Vol. 6. Owl Publishing House Co., Ltd., Taipei.

Hoch PC, Gandhi K (2020) Nomenclatural changes in Onagraceae. Phytokeys (accepted)

Repin R, Sampang K, Limbawang S. (2015). *Begonia* (Begoniaceae) from Tawau Hills Park, Sabah, Malaysia, including ten new species. Sandakania 20:165–204.
