# Supplementary material for: In memoriam Ching-I Peng (1950–2018)—an outstanding scientist and mentor with a remarkable legacy
Source: Bot Stud. 2020 Apr 25;61:14. doi: 10.1186/s40529-020-00291-5 (PMC7182648; doi:10.1186/s40529-020-00291-5)
Supplement: Supplementary file 4 — Additional file 4. Ching-I Peng’s contribution to the flora of Taiwan: new distribution records (71 species). [file 40529_2020_291_MOESM4_ESM.pdf]

Additional file 4. Ching-I Peng's contribution to the flora of Taiwan: new distribution records (71 species). See Additional file 2 for relevant reference (superscripts following the Chinese plant names).

Araceae (1)

1. *Amorphophallus kiusianus* (Makino) Makino 東亞魔芋 [50]

Asteraceae (32)

2. *Acmella brachyglossa* Cass. 短舌花金鈕扣 [151]
3. *Acmella ciliata* (Kunth) Cass. 天文草 [151]
4. *Ageratina adenophora* (Spreng.) R.M.King & H.Rob. 假藿香薊（紫莖澤蘭）[65]
5. *Ageratina riparia* (Regel) R.M. King & H.Rob. 澤假藿香薊 [168]
6. *Ambrosia psilostachya* DC. 裸穗豬草 [111]
7. *Artemisia chingii* Pamp. 南毛蒿 [72]
8. *Artemisia simulans* Pamp. 中南蒿 [72]
9. *Aster subulatus* Michaux var. *sandwicensis* (A.Gray) A.G.Jones 澤掃帚菊 [72]
10. *Austroeupatorium inulifolium* (Kunth) R.M.King & H.Rob. 假澤蘭 [132]
11. *Blumea manillensis* DC. [= *Blumea lanciata* (Roxb.) DC.] 旗山艾納香 [4]
12. *Calyptocarpus vialis* Less. 金腰箭舅 [11]
13. *Chromolaena odorata* (L.) R.M.King & H.Rob. 香澤蘭(飛機草) [64]
14. *Cichorium intybus* L. 菊苣 [4]
15. *Clibadium surinamense* L. 蘇利南野菊 [155]
16. *Cotula australis* (Sieber ex Spreng.) Hook.f. 南方山芫荽 [168]
17. *Emilia praetermissa* Milne-Redh. 粉黃纓絨花 [174]
18. *Emilia sagittata* (Vahl.) DC. [= *Emilia fosbergii* Nicolson] 纓絨花 [4]
19. *Erigeron bellioides* DC. 類雛菊飛蓬 [168]
20. *Flaveria bidens* (L.) Kuntze 黃頂菊 [164]
21. *Flaveria linearis* Lag. 線葉黃頂菊 [208]
22. *Galinsoga quadriradiata* Ruiz & Pav. 粗毛小米菊 [65]
23. *Gnaphalium luteoalbum* L. 絲綿草 [72]
24. *Helianthus debilis* Nutt. subsp. *cucumerifolius* (Torrey & A.Gray) Heiser 瓜葉向日葵 [159]
25. *Hypochaeris radicata* L. 貓兒菊 [4]
26. *Parthenium hysterophorus* L. 銀膠菊 [32]
27. *Petasites japonicus* (Siebold & Zucc.) Maxim. 蜂斗菜 [4]
28. *Pluchea carolinensis* (Jacq.) G.Don 美洲闊苞菊 [62]
29. *Pluchea sagittalis* (Lam.) Cabera 翼莖闊苞菊 [62]
30. *Pyrrhopappus carolinianus* (Walter) DC. 大蒲公英舅 [274]
31. *Soliva pterosperma* (Juss.) Less. 翅果假吐金菊 [44]
32. *Taraxacum officinale* F.H.Wigg. 西洋蒲公英 [4]
33. *Vernonia elliptica* DC. 光耀藤 [65]

Begoniaceae (1)

34. *Begonia grandis* Dry. 秋海棠 [244]

Brassicaceae (2)

35. *Arabis serrata* Franch. & Sav. 齒葉南芥 [52]

36. *Lepidium bonariense* L. 南美獨行菜 [120]

Campanulaceae (2)

37. *Lobelia cliffortiana* L. 克氏半邊蓮 [191]

38. *Triodanis biflora* (Ruiz & Pav.) Greene 卵葉異擔花 [58]

Chloranthaceae (1)

39. *Choranthus henryi* Hemsl. 寬葉金粟蘭 [138]

Commelinaceae (2)

40. *Cyanotis axillaris* (L.) Sweet 鞘苞花 [85]

41. *Murdannia spirata* (L.) Brückner 矮水竹葉 [22]

Euphorbiaceae (1)

42. *Croton bonplandianus* Baillon 波氏巴豆 [129]

Fabaceae (4)

43. *Alysicarpus rugosus* (Willd) DC. 皺果煉莢豆 [19]

44. *Medicago arabica* (L.) Huds. 褐斑苜宿 [200]

45. *Medicago minima* (L.) Bartal. 小苜蓿 [200]

46. *Mimosa pigra* L. 刺軸含羞草 [93]

Geraniaceae (1)

47. *Geranium carolinianum* L. 野老鸛草 [4]

Loganiaceae (1)

48. *Gardneria nutans* Siebold & Zucc. 垂花蓬萊葛 [46]

Melanthiaceae (1)

49. *Ypsilandra thibetica* Franch. 丫蕊花 [190]

Onagraceae (5)

50. *Ludwigia adscendens* (L.) H.Hara 白花水龍 [7]

51. *Ludwigia decurrens* Walter 翼莖水丁香 [183]

52. *Ludwigia erecta* (L.) H.Hara 美洲水丁香 [183]

53. *Ludwigia palustris* (L.) Elliott 沼生水丁香 [183]

54. *Oenothera laciniata* Hill 裂葉月見草 [17]

Orobanchaceae (1)

55. *Phacellanthus tubiflorus* Siebold & Zucc. 黃筒花 [186]

Plantaginaceae (1)

56. *Veronica hederifolia* L. 睫毛婆婆納 [166]

Polygonaceae (1)

57. *Persicaria capitata* (Buch.-Ham. ex D.Don) H.Gross 頭花蓼 [115]

Rosaceae (1)

58. *Rubus amphidasys* Focke 周毛懸鉤子 [142]

Rubiaceae (3)

59. *Oldenlandiopsis callitrichoides* (Griseb.) Terrell & W.H.Lewis 匍匐微耳草 [193]

60. *Spermacoce assurgens* Ruiz & Pavon 光葉鴨舌廣舅 [23]

61. *Spermacoce pusilla* Wall. 小鴨舌癀舅 [27]

Smilacaceae (1)

62. *Heterosmilax septemnervia* F.T.Wang & Tang 短柱土茯苓 [196]

Solanaceae (7)

63. *Capsicum annuum* L. 辣椒 [71]

64. *Datura inoxia* Mill. 毛曼陀羅 [71]

65. *Lycopersicon esculentum* var. *cerasiformis* (Dunal) A.Gray 櫻桃小番茄 [71]

66. *Physalis pubescens* L. 毛酸漿 [71]

67. *Solanum pseudocapsicum* L. 珊瑚櫻 [71]

68. *Solanum scabrum* Miller 木龍葵 [71]

69. *Solanum seaforthianum* Andrews 星茄 [71]

Verbenaceae (1)

70. *Verbena bonariensis* L. 柳葉馬鞭草 [16]

Violaceae (1)

71. *Hybanthus enneaspermus* (L.) F.Muell. 鼠鞭草 [12]
